# Splicing-Related Features of Introns Serve to Propel Evolution

**DOI:** 10.1371/journal.pone.0058547

**Published:** 2013-03-13

**Authors:** Yuping Luo, Chun Li, Xi Gong, Yanlu Wang, Kunshan Zhang, Yaru Cui, Yi Eve Sun, Siguang Li

**Affiliations:** 1 Stem Cell Translational Research Center, Tongji Hospital, Tongji University School of Medicine, Shanghai, China; 2 College of Life Sciences, Nanchang University, Nanchang, China; 3 Shanghai Stem Cell Institute, Institutes of Medical Sciences, Shanghai Jiao Tong University School of Medicine, Shanghai, China; CNRS UMR7275, France

## Abstract

The role of spliceosomal intronic structures played in evolution has only begun to be elucidated. Comparative genomic analyses of fungal snoRNA sequences, which are often contained within introns and/or exons, revealed that about one-third of snoRNA-associated introns in three major snoRNA gene clusters manifested polymorphisms, likely resulting from intron loss and gain events during fungi evolution. Genomic deletions can clearly be observed as one mechanism underlying intron and exon loss, as well as generation of complex introns where several introns lie in juxtaposition without intercalating exons. Strikingly, by tracking conserved snoRNAs in introns, we found that some introns had moved from one position to another by excision from donor sites and insertion into target sties elsewhere in the genome without needing transposon structures. This study revealed the origin of many newly gained introns. Moreover, our analyses suggested that intron-containing sequences were more prone to sustainable structural changes than DNA sequences without introns due to intron's ability to jump within the genome via unknown mechanisms. We propose that splicing-related structural features of introns serve as an additional motor to propel evolution.

## Introduction

Spliceosomal introns, one of the hallmarks of eukaryotic genomes, exist in eukaryotic protein-coding genes [1]–[4] and non-protein-coding genes [5]–[6]. After transcription, they are inevitably removed from corresponding RNA transcripts. Although more than thirty years have passed since the discovery of spliceosomal introns, fundamental questions about spliceosomal intron evolution, including evolutionary origins of introns, their ages, natural selection pressures imposed on them, as well as how introns are lost and gained during evolution, have only begun to be elucidated. Previous studies of intron loss and gain mainly focused on protein-coding genes [7]–[10], which are subjected to huge natural selection pressure because small changes in nucleotide sequences in exons (lost or gained) tend to drastically alter protein structures and functions. As a result, many alterations in protein encoding genes could not survive or leave traces during evolution, rendering previous studies on protein-encoding genes incapable of revealing the full spectrum of changes in intron dynamics during evolution.

Several lines of evidence suggest that half or more of mammalian transcriptomes consist of non-coding RNAs (ncRNAs), many of which are subjected to splicing [11]–[13]. Non-coding RNA genes are under less stringent selection pressure than protein-coding genes because their functional units are in general short and their other parts are easier to tolerate sequence alterations [14]. On the other hand, the presence of conserved non-coding RNAs in introns of non-coding genes has facilitated tracking of intron loss and gain events. snoRNA, an abundant class of non-protein-coding RNAs, is widespread in eukaryotes from yeast to human. Because snoRNAs in gene clusters detected so far in fungi are conserved and encoded by independent genes or nested within introns of non-protein-coding host genes, it is appropriate to use snoRNA gene clusters as evolutionary conserved marks to track exon/intron modifications during evolution. Recently, by comparing conserved snoRNAs in introns, an alternative mechanism for intron loss through widespread degeneration of splicing signals (de-intronization) was uncovered in *Saccharomyces* [15], demonstrating the power of studying intron loss and gain in non-coding RNA genes.

In this study, we performed systematic analysis of intron and exon architecture of noncoding snoRNAs from available fungal genome databases. We analyzed all noncoding snoRNA sequences from multiple complete genome sequences and high-quality draft sequences and compared intron presence/absence polymorphisms among these fungi. Our analyses revealed intron loss and gain events and possible underlying evolution mechanisms in three conserved snoRNA gene clusters. In addition, we verified splicing patterns for complex introns (several introns lie in juxtaposition without intercalating exons) derived from internal exon loss by applying systems biology tools and experimentation. Finally, we have found snoRNA genes located within intronic sequences could move around within the genome. Our analyses suggest that intronic structures are more prone to translocation via unknown mechanisms. Based on these findings we propose a novel evolution mechanism, i.e., intronic structures serve as an additional motor to propel evolution.

## Results

### Intron distributions within three snoRNA gene clusters in fungi

**Figure 1:**
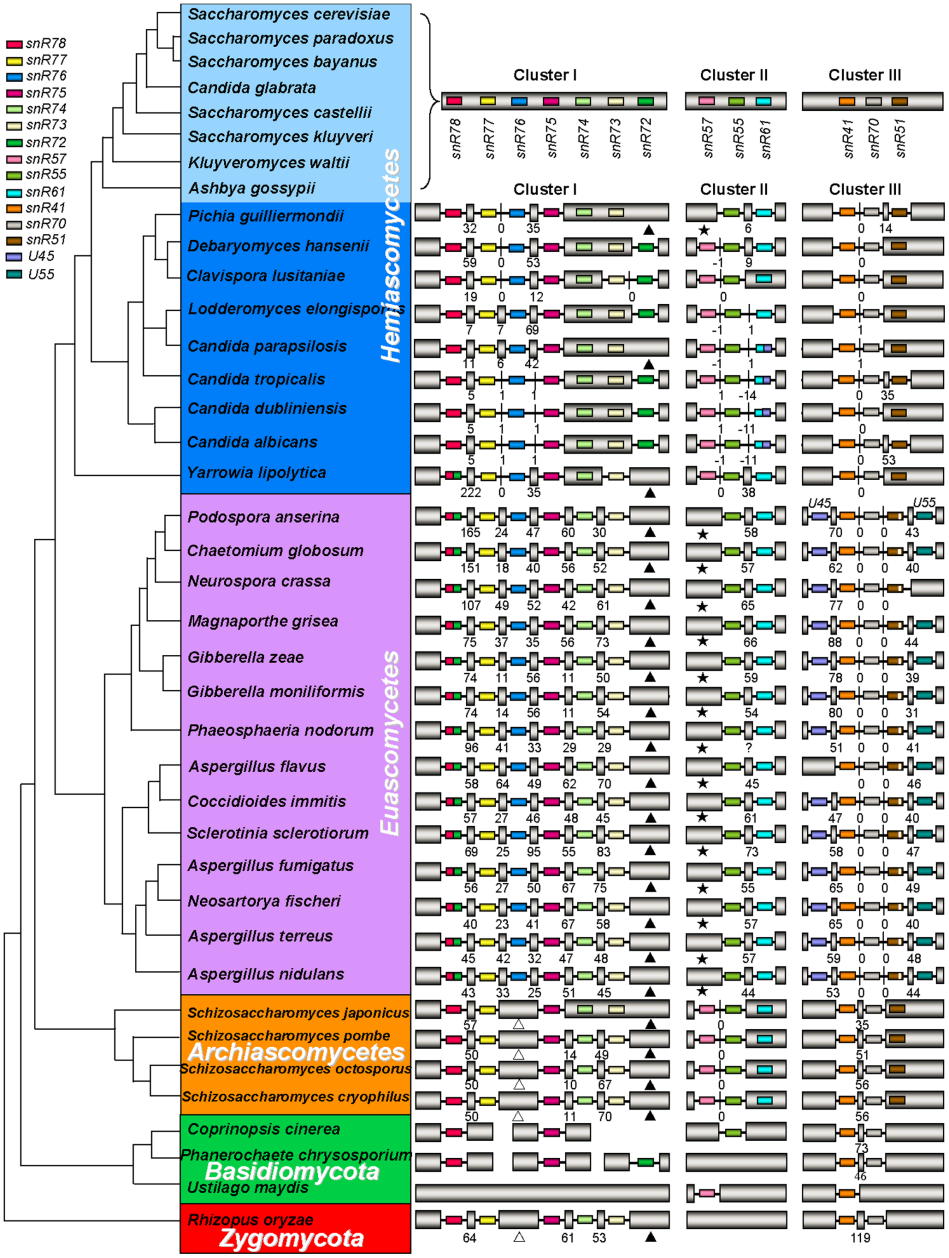
The distribution of snoRNA-associated introns in fungal Polycistronic snoRNA gene cluster I, II and III. Cladogram showing *Basidiomycota* (green), *Zygomycota* (red) and *Ascomycota*, subdivided into *Hemiascomycetes* (blue), *Euascomycetes* (purple), and *Archiascomycetes* (orange). snoRNA gene clusters I, II and III exist widespread in fungi genomes. The location where the snR76-, snR72- and snR57-associated intron were lost are marked with open triangle, filled triangle and filled star, respectively. snoRNAs are represented schematically by different colored boxes, introns as lines, and exons as gray pillars, with internal exons labeled by size. In detail: cluster I, snR78-snR77-snR76-snR75-snR74-snR73-snR72; cluster II, snR57-snR55-snR61; cluster III, snR41-snR70-snR51. All are drawn not to scale.

In order to acquire insight into the evolutionary dynamics of introns in fungal non-coding RNA, we used multiple complete genome sequences and high-quality draft sequences in fungi as references for our analyses and systematically analyzed three snoRNA gene clusters, named snoRNA gene cluster I, II and III, which encode snR78-snR77-snR76-snR75-snR74-snR73-snR72, snR57-snR55-snR61 and snR41-snR70-snR51 snoRNAs, respectively. We found that nearly all analyzed fungi have these snoRNA cluster sequences. The structure features of these snoRNAs are such that they possess box C (5'-TGATGA-3') and D (5'-CTGA-3' or 5’-ATGA-3’) near their 5' and 3' ends together with one or two functional elements involved in guiding 2'-O-ribose methylation of rRNA [16]. We searched for splicing signals in sequences adjacent to snoRNAs in these clusters, and predicted 323 intronic snoRNAs from 460 analyzed snoRNAs (Figure 1), among which we have verified 83 of these introns by comparison of expressed sequence tags (ESTs) from these species to the corresponding genomic sequences and/or by reverse transcription polymerase chain reaction (RT-PCR) and sequencing (Table S1,S1, S2, S3). Although there is not enough EST data to verify the rest intronic snoRNAs, they have canonical 5’ splice sites, canonical 3’ splice sites and branch point sequences of intron and therefore are indeed intronic. The spliced RNAs from these fungal polycistronic snoRNA host genes, similar to spliced mammalian U22 snoRNA host gene RNA [17], are poorly conserved and lack long open reading frames, therefore having little potential for protein coding.

In these three clusters, intron distribution among different fungal groups varies considerably. In *Euascomycetes* of *Ascomycota* (the species labeled in purple in Figure 1), *Basidiomycota* (the species labeled in green in Figure 1) and *Zygomycota* (*Rhizopus oryzae*), snoRNAs in these three clusters are all intronic; in some species of *Hemiascomycetes* except for *Saccharomyces cerevisiae* and its close relatives (the species labeled in dark blue in Figure 1) and *Archiascomycetes* (the species labeled in orange in Figure 1) most of them are within individual introns; whereas in *S. cerevisiae* and its close relatives (the species labeled in baby blue in Figure 1), all of them arise from unspliced primary transcripts. This is presumably correlated with intron densities within the genome among different fungal species, since it is reported that at least for protein-encoding genes, the intron density is 2–3 introns/gene for *Euascomycetes* fungi *Neurospora crassa* and *Aspergillus nidulans* [18]–[20], 0.9 intron/gene for *Archiascomycetes* fungi *Schizosaccharomyces pombe* [21], and 0.05 intron/gene [22] for *Hemiascomycetous* fungi *S. cerevisiae*.

### Intron presence/absence polymorphisms resulting from intron loss and gain events

Previous analyses of eukaryotic genomes revealed that the common ancestor of fungi and animals was intron-rich [23], [24]. Thus, extensive ancestral intron loss was suggested to account for intron-poor eukaryotes. The current non-intronic snoRNA gene cluster I, II and III in *S. cerevisiae* and its close relatives should result from intron loss events through splice-site degeneration mechanisms (de-intronization of intronic sequences) during fungi evolution (Figure 1), as proposed recently for non-coding RNA introns [15]. After de-intronization of snoRNA-associated intronic sequences, the snoRNAs in these three intron-less clusters in *S. cerevisiae* and its close relatives remain stable in sequence structure, including snoRNA number and order.

**Figure 2:**
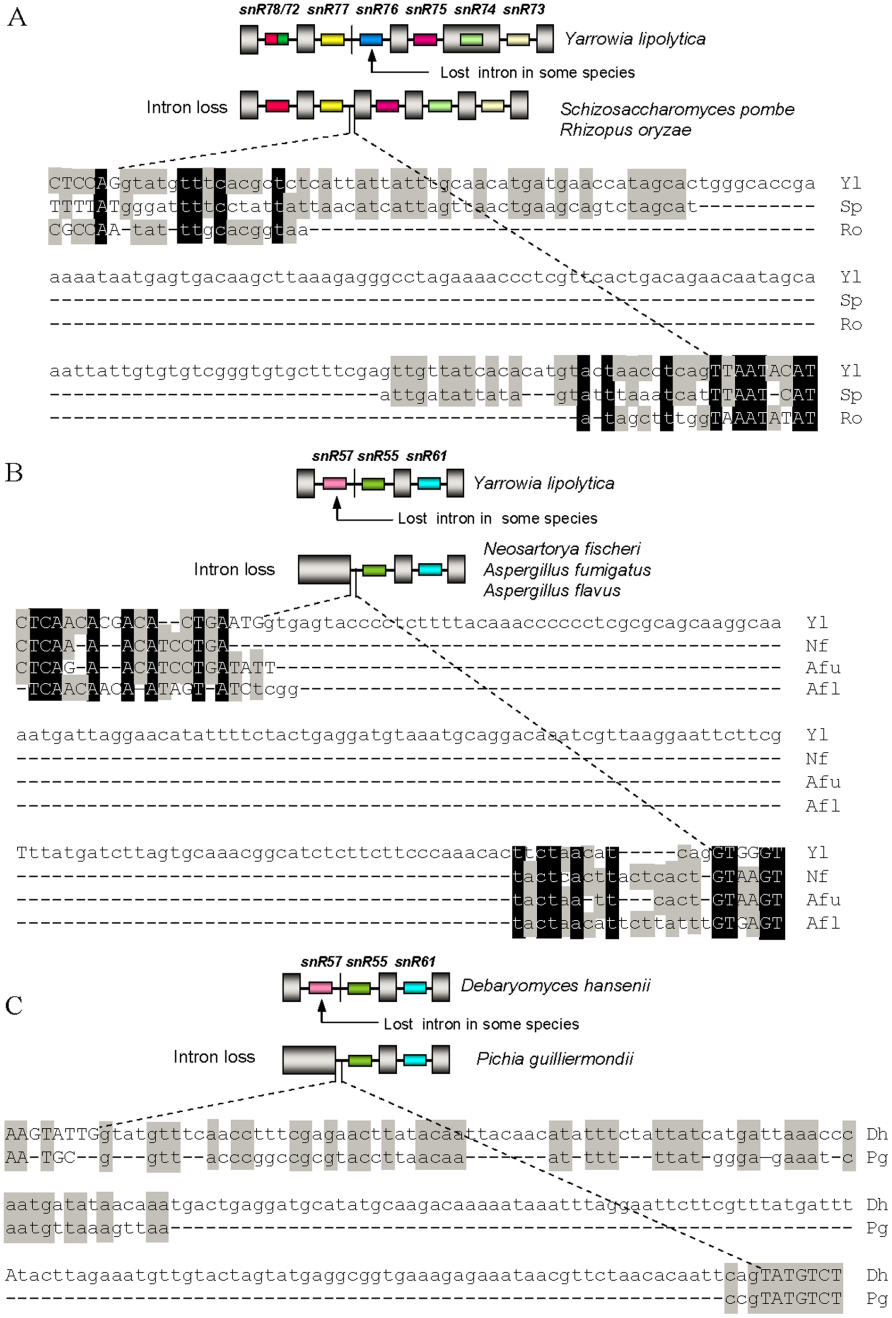
Examples of intron loss by genomic deletion. (*A*) Genomic deletion of most of an intron sequence which harbor snR76 from snoRNA cluster I in *Schizosaccharomyces pombe* (Sp) and *Rhizopus oryzae* (Ro), respectively. (*B*) Genomic deletion of most of an intron sequence which harbor snR57 from snoRNA cluster II in *Neosartorya fischeri* (Nf), *Aspergillus fumigatus* (Afu), *Aspergillus flavus* (Afl), *Pichia guilliermondii* (Pg), respectively. (*C*) Genomic deletion of most of an intron sequence which harbor snR57 from snoRNA cluster II in *Pichia guilliermondii* (Pg). snoRNAs are represented schematically by different colored boxes, introns as lines or in lowercase letters, and exons as gray pillar or in capital letters. Conserved sequences are shaded. All are drawn not to scale.

However, one third of snoRNA-associate introns in intronic snoRNA gene clusters show polymorphisms (Figure 1) among analyzed fungi. SnR76-associated introns in cluster I of all the *Archiascomycetes* species and *R. oryzae* (marked with open triangle in Figure 1), snR72-associated introns in cluster I of most fungal species (marked with filled triangle in Figure 1), as well as snR57-associated introns in cluster II of all *Euascomycetes* species and *Pichia guilliermondii* (marked with filled star in Figure 1) were lost together with their intronic snoRNAs from snoRNA gene clusters, respectively. Sequence alignment showed that elimination of snR76-associated and snR57-associated introns possesses characteristics of the “genomic deletion model” postulated a while ago for intron loss in protein-coding genes [10]. Those characteristics include retention of residual intron sequences and a lack of biased loss of 3’ introns. These intron loss cases clearly result from genomic deletion events followed by subsequent divergence of remainder intronic sequences (Figures 1, 2). In addition, besides intron loss, we also observed intron gain events. U45-associated and U55-associated introns were inserted in snoRNA gene cluster III at both ends in nearly all the *Euascomycetes* species (species highlighted in purple in Figure 1). These intron presence/absence polymorphisms in intronic snoRNA gene clusters likely result from intron loss and gain events during fungi evolution, based on the presence or absence of introns in homologous positions of orthologous genes of widely divergent fungi that we observed (Figure 1).

### Evidence for the origin of recently gained introns

**Figure 3:**
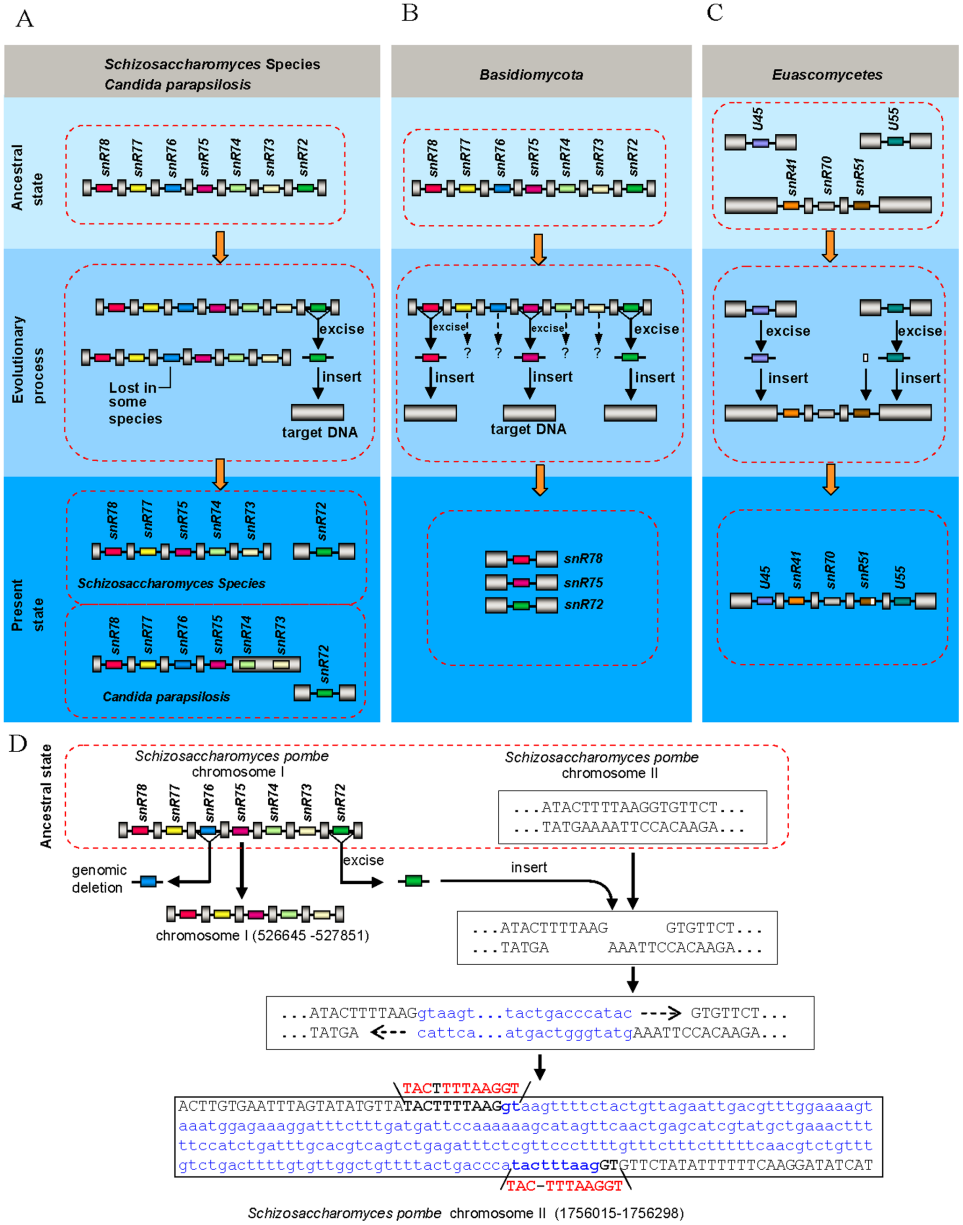
“excised and inserted” pattern for intron loss and gain. (*A*) SnR72-associated intron loss and gain in *Schizosaccharomyces* species and *Candida parapsilosis*. (*B*) SnR78, snR75 and snR72-associated intron loss and gain in *Basidiomycota*. Question marks indicate snoRNA-associated introns which are not found in *Basidiomycota*. (*C*) U45 and U55-associated intron loss and gain in *Euascomycetes*. (*D*) An example of intron loss and gain by the mechanism of “excised and inserted”. DNA insert site is show in capital letters, inserted sequence is show in lowercase letters and short direct repeats are marked in red.snoRNAs are represented schematically by different colored boxes, introns as lines, and exons as gray pillars. All are drawn not to scale.

By tracking snoRNAs in cluster I, II and III in different fungal species, we found some intron gain events that are linked to intron loss events. In *Schizosaccharomyces* species and some *Candida* species, the snR72-associated introns were lost in the cluster I sites, but reappeared in other places within the genome (Figure 3A). In addition, snR78, snR75 and snR72-associated introns in some species of *Basidiomycota* are no longer located in the snR72–78 poly-cistronic cluster, instead, they scatter over different sites in the genome (Figure 3B). Moreover, the U45 and U55-associated intron disappear from original sites and are inserted into snR41-snR70-snR51 poly-cistronic gene cluster in most species of *Euascomycetes* (Figure 3C), which provided direct evidence for the origin of some newly gained introns.

How were these snoRNA-associated introns lost from donor sites and reinserted into target sites? Currently, there are several main models for intron loss and gain. Reverse-transcriptase-mediated 3’-biased intron loss model [10] and genomic deletion model [10] were proposed as mechanisms underlying some permanent intron loss from genome. The model of degeneration of splicing signals [15] has been used to explain de-intronization of intronic sequences. On the other hand, intron transposition [10], [25], [26], self-splicing type II intron [10], [27], [28], and genomic duplication [10] have been postulated to explain different intron-gain events, in which newly gained introns are inserted into somewhere else in the genome with the original sequences remaining in the donor sites. New introns may also arise from repair of staggered double-strand breaks (DSBs) accompanied by small segmental insertions, however the origins of those newly gained introns are unclear [29]. Only the intron transfer model deals with introns loss from donor sites and become gained in the target sites. However, according to the model, intron transfer may only occur between paralogous [10], [25], [26]. Taken together, currently no single model offers clear explanations for both loss and gain of these snoRNA-associated introns. Given the loss of some snoRNA-associated introns from their donor sites and insertion into target sites with no homology between donor and recipient genes (Figure 3) and given that there exists short direct repeats (Figure 3D, Table S2) within some gained snoRNA-associated introns, we proposed a new model, named “excision-and-insertion” model, for intron loss and gain, i.e., they are excised from the donor sites (Figure 3) as complete or nearly complete intron units at the DNA level and got inserted into staggered double-strand breaks sites of the genome. Our results suggested that some introns might move from site to site within the genome without harboring transposon structures.

**Figure 4:**
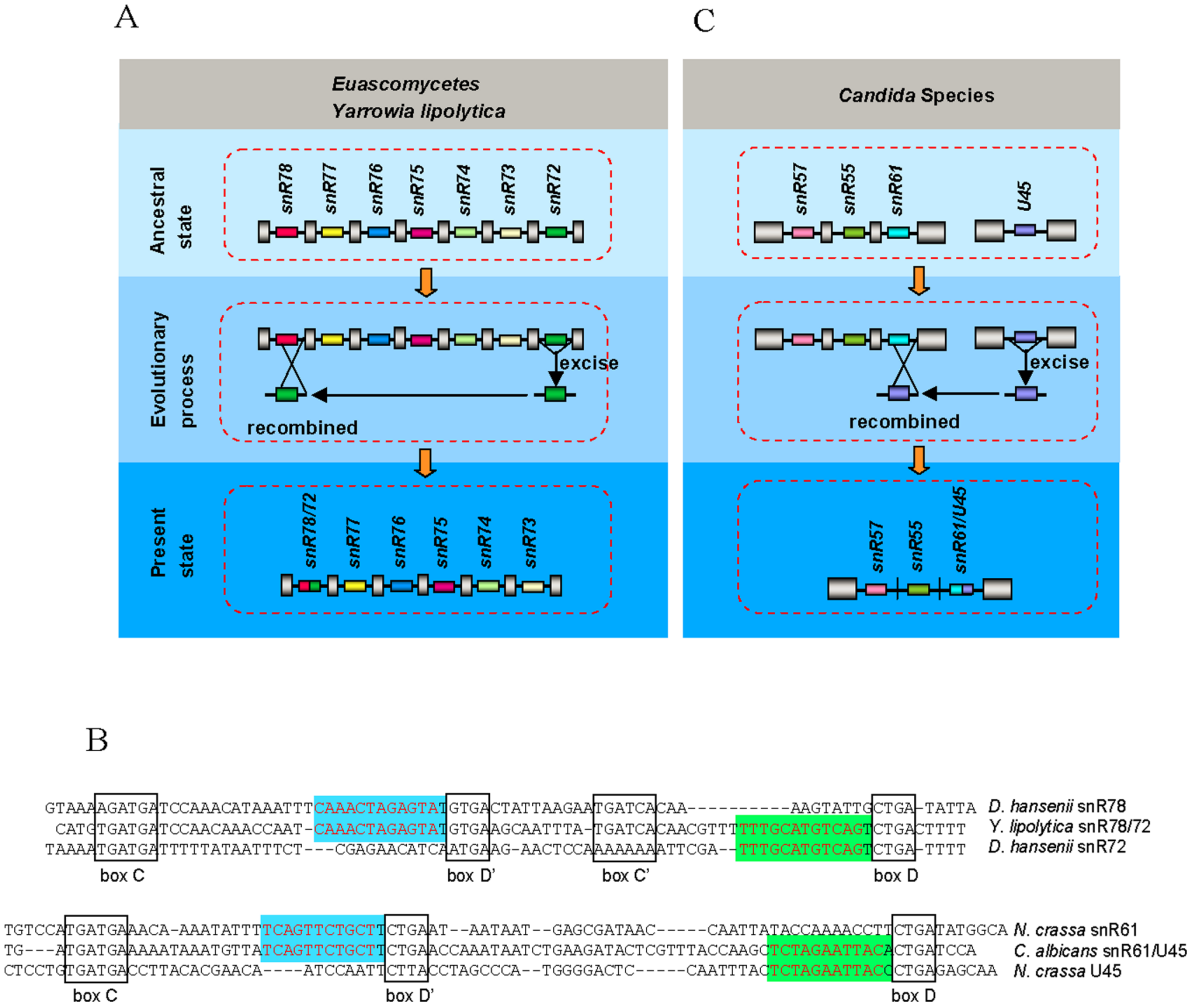
Recombination of snoRNA-associated intron. (*A*) Recombination between snR72-associated intron and snR78-associated intron in *Euascomycetes* and *Yarrowia lipolytica*. snoRNAs are represented schematically by different colored boxes, introns as lines, and exons as gray pillars. All are drawn not to scale. (*B*) The sequences of snR78, snR72, snR78/72, snR61, U45 and snR61/U45. Conserved motifs C, D′, C′ and D are indicated by boxed sequences. The functional sequence complementarity to the rRNA is in red. (*C*) Recombination between U45-associated intron and snR61-associated intron in *Candida* Species. snoRNAs are represented schematically by different colored boxes, introns as lines, and exons as gray pillars. All are drawn not to scale.

Obviously, not all excised snoRNA-associated introns could be inserted directly into the genome. What's interesting is that some of the excised introns can recombine with other sequences to form reorganized introns. snR78/72-associated intron in all species of *Euascomycetes* fungi and one species of *Hemiascomycetous* fungi (*Y. lipolytica*) came from recombination between snR72-associated intron and snR78-associated intron (Figure 1, Figure 4A, 4B), which is a good indication for “reorganized intron evolution” event. The snR61/U45-associated intron structure in *Candida* species indicates an additional example of such evolution mechanism (Figure 1, Figure 4B, 4C). Finally, the snR73, snR74, snR76 and snR77-associated introns in some species of *Basidiomycota* fungi (Figure 3B) and U45 and U55-associaed introns in all fungi except for *Euascomycetes* (Figure 1) disappeared from the genome, suggesting another alternative fate of excised snoRNA-associated introns, which is permanent elimination from the genome. Together, these observations indicate that excised snoRNA-associated introns have three different fates: direct reinsertion into elsewhere within the genome (Figure 3), recombination with other sequences (Figure 4) or lost from genome (Figure 3B).

**Figure 5:**
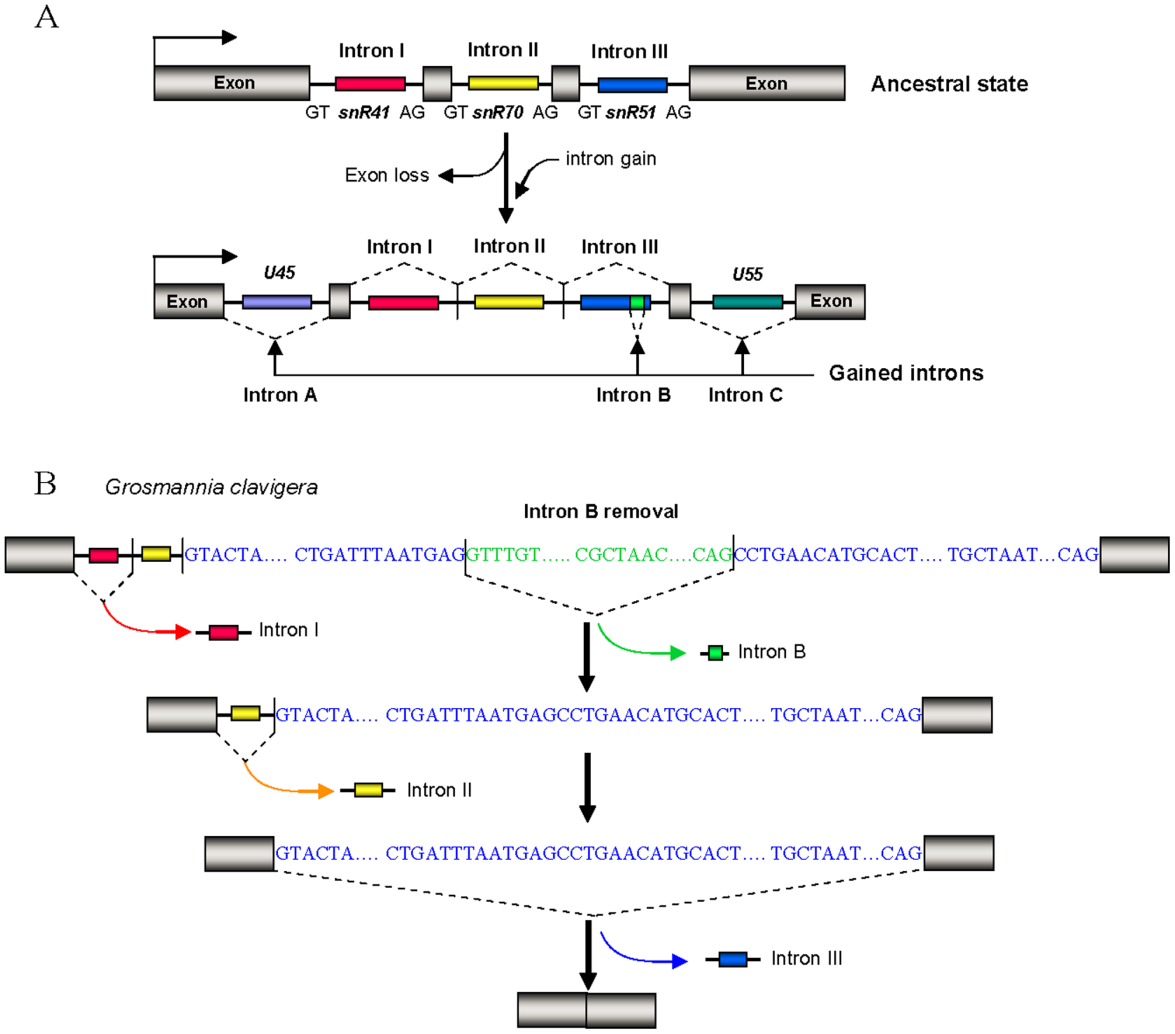
Intron gain and exon loss and splicing in *Euascomycetes* snR41-snR70-snR51 polycistronic cluster III. (*A*) Intron gain and exon loss in *Euascomycetes* snR41-snR70-snR51 polycistronic cluster III. snoRNAs are represented schematically by different colored boxes, introns as lines, and exons as gray pillars. (*B*) Stepwise splicing of snR41-snR70-snR51 polycistronic cluster in *Euascomycetes*. snoRNAs are represented schematically by different colored boxes, introns as lines, and exons as gray pillars. The 5′ splice site, branch site and 3′ splice site sequences are shown in blue letters for intron III and green letters for intron B.

Besides short direct repeats mentioned above, we also observed short inverted repeats, ranging in size from 8 to 12 base pairs, with one repeat positioned within the end of an adjacent exon sequence and the other repeat near the opposite end of the inserted intron (Figure S4A). In order to know whether this kind of short inverted repeats exist in gained non snoRNA-associated intron, we analyzed a gained intron (intron B) which is inserted into intronic snR51 of cluster III at the position between conserved antisense functional sequence involved in guiding 2'-O-ribose methylation of rRNA and conserved box D (Figure 5A). In different species of *Euascomycetes* fungi, the inserted intron sequences are significantly divergent (Figure S5), and the surrounding exons demonstrate similar divergence (Figure S6), suggesting that the intron-gain event appears to have occurred in the ancestor of the *Euascomycetes* and may be a single intron-gain event followed by subsequent divergent changes of intronic sequences. After careful analysis of the inserted intron B and its flanking sequences, we found that intron B in the ancestor of the *Euascomycetes* and its flanking exons (conserved antisense functional sequence of snR51) also harbored short inverted repeats (Figure S4B). Moreover, we found this kind of inverted repeats also existed in recently gained introns resulting from repair of double-strand breaks (DSBs) accompanied by small segmental insertions in *D. pulex* protein coding genes [29] (Figure S4C), suggesting these short inverted repeats are likely related to the novel intron loss-gain event mentioned above.

### Exon loss and splicing of complex introns

Previous work confirmed that fungal introns in protein encoding gene are typically short and exons are long relative to their mammalian counterparts [30], but recent work showed that some internal exons within polycistronic snoRNA gene clusters are small, even absent in *Candida* clade and more distantly related *Hemiascomycete Y. lipolytica* (15). The genomic deletion of internal exons in polycistronic snoRNA gene clusters lead to complex intron architectures, where several introns lie in juxtaposition without intercalating exons. Interestingly, we found that such complex introns are not specific to *Candida* clades and *Y. lipolytica* of *Hemiascomycetes*, but also exist in *Euascomycetes* and *Archiascomycetes* (Figure 1), suggesting exon loss could be a common phenomenon in fungi evolution. Taken together, we found at least 61 exon loss events in these clusters in *Hemiascomycetes, Euascomycetes* and *Archiascomycetes* fungi (Table S3). Because much of the available genomic information is still incomplete, our results may only represent a subset of such complex introns in fungi.

The traditional splicing of spliceosomal introns is mediated by the spliceosome, which interacts with specific parts of the intron and the flanking exons to ensure accurate and efficient splicing [31]. Because these complex introns analyzed above are novel constitutions of eukaryotic gene, their splicing characteristics are still unknown. To track their splicing pattern, we systematically analyzed spliced products of polycistronic snoRNA precursors from cluster III in *Euascomycetes* fungal species. Polycistronic snoRNA gene cluster III in all *Euascomycete* species misses two interior exons, forming an “intron-intron-intron” structure (Figure 1, 5A). In addition, two snoRNA-associated introns, U45 and U55-associated introns (intron A and intron C) and one non-snoRNA-associated intronic sequence (intron B) are inserted in snoRNA gene cluster III (Figure 5A). We have compared expressed sequence tags (ESTs) from EST database of *Euascomycetes* fungal species to their corresponding genome sequences and found 42 spliced products from cluster III transcripts. Among the 42 spliced products, 18 removed intron I, but retained intron II and III with their intronic snoRNA; 14 removed intron I and II, but retained intron III with their snoRNA; 10 removed all the three introns (Figure S7, S8, S9, S10, S11, S12, S13, S14).

We did not detect any spliced products that result from removal of intron II and/or intron III but retaining intron I or removal of intron III but retaining intron I and/or intron II. This indicates that intron I, II and III may be removed by stepwise splicing from the 5’ end to the 3’ end of the transcripts (Figure 5B), i.e., intron I was preferentially removed at first splicing step, and splicing of intron II can occur only after intron I has been removed, and followed by the removal of intron III. Alternatively, it is also possible that only intron I, which is next to an exon, contains a functional splicing donor, and this donor may pick any of the three functional splicing acceptors in the 3’end of each intron to conduct splicing, resulting in the products we detected.

To further verify the stepwise splicing pattern or alternative acceptor usage for such unusual introns, we performed RT-PCR amplification with YLC2F1/YLC2R1 primer pair for *Y.lipolytica* cluster II, DhC2F1/DhC2R1 and DhC3F1/DhC3R1 primer pairs for *Debaryomyces hansenii* cluster II and III, respectively. We then cloned the RT-PCR products, sequenced them, and analyzed spliced products. Our result demonstrated that the unusual introns of cluster II and III in *D. hansenii* and *Y. lipolytica* could potentially be stepwise spliced from the 5’ end to the 3’ end (Figure S1, S2, S3) or spliced via alternative splicing acceptor usage mentioned above. As expected, we couldn't get any splice products containing intron I without intron II and/or intron III. This splicing pattern was further confirmed by additional RT-PCR analyses with specialized primer pairs DhC2F2/DhC2R1 and YLC2F2/YLC2R1, again, the first removal of intron II and/or intron III could never be detected. Such splicing patterns suggest that, consistent with intron splicing in protein encoding genes [31], 5’ exon sequences are required to ensure functionality of splicing donors to perform accurate and efficient splicing for non-coding RNA gene.

## Discussion

We have performed systematic analysis of intron and exon architecture of three noncoding snoRNA gene clusters from available fungal genome databases and found that intron distributions in non-protein-coding genes among different fungal groups vary considerably (Figure 1). In three snoRNA gene clusters, all of the snoRNAs in *S. cerevisiae* and its close relatives reside in unspliced primary transcripts (Figure 1), potentially resulting from substantial intron loss via degeneration of their splicing signals [15]. After de-intronization of intronic sequences, the snoRNAs in the three intron-less clusters in *S. cerevisiae* and its close relatives remain stable in sequence structure. However, one third of intronic snoRNAs in other fungi show presence/absence polymorphisms due to intron loss and gain events during evolution (Figure 1). In addition, the intronic snoRNA gene clusters had also experienced exon loss and we found 61 exon loss cases in *Hemiascomycetes*, *Euascomycetes* and *Archiascomycetes* fungi (Table S3). The presence of complex introns where multiple introns reside in juxtaposition is a clear indication for exon loss. Taken together, it appears that intron-containing sequences are more prone to structural changes than sequences without introns. Therefore splicing-related features of introns may serve as an additional motor to propel evolution, though how RNA splicing machinery influences excision of DNA elements remained to be determined.

Analysis of the ultimate fate of the excised snoRNA-associated introns suggests that intron loss events could be independent in different lineages. For example, in *Archiascomycetes* fungi, the snR72-associated introns were inserted as a whole into target sites of genome, whereas in *Euascomycetes* fungi, the snR72-associated introns recombined with other sequences to form reorganized introns (Figure 1, Figures 3, 4), suggesting the excision of snR72-associated introns happened after the divergence of *Archiascomycetes* fungi and *Euascomycetes* fungi and therefore were obviously independent events. This suggests that some intron sequences might be hot spots for excision.

How introns spread within the genome remains an unanswered question in evolution biology [5]. Identifying the origin of recently gained introns is likely a key to understanding where new introns come from. For recently gained introns in *Caenorhabditis elegans* and *Caenorhabditis briggsae*, reverse splicing of preexisting introns [28] is the main mechanism for intron gain during recent nematode evolution [32]. However, studies of protein-coding genes in *Daphnia* population revealed that more than half of the recent gained introns were associated with short sequence repeats, which were formed via repair of staggered double-strand breaks. However, the sources of these gained introns, except for one, still remain unknown [29]. By tracking conserved snoRNAs in introns, we found that the gained introns by repairing double-strand breaks are derived from excised introns from other sites. The failure of previous studies to find the sources of recently gained introns in *Daphnia* population can be explained by the fact that there are no conserved sequences within these introns, making it difficult to track their origins. Our study demonstrated that intron loss and gain by a mode of excision from donor sites and reinsertion into the target sites (Figure 1, 3) may represent a novel mechanism underlying exon-intron structure evolution.

Besides short direct repeats mentioned above, we also found short inverted repeats associated with some recently gained snoRNA-associated introns (Figure S4A). These short inverted repeats differ from the inverted repeats of transposons. Transposons consist of inverted repeats at both ends, which are recognized by transposase, followed by excision and re-insertion into a new location [32], [33]. However, in some recently gained introns in this work, the short inverted repeats exist in target site and inserted exogenous DNA fragment, respectively. The function of the short inverted repeats remains to be revealed. We hypothesize that the short inverted repeats may act as sequence-specific guides for recognition between excised intron sequence and target sequence via base pairing. If the existence of short inverted repeats acts as guide for interactions between excised intron and target sequence, we would predict that intron removal from one location and insertion into another is site-specific, rather than random.

DNA sequences should have been subjected to enormous alterations during evolution. However, due to the presence of natural selection, many genomic alterations are erased without leaving a trace. snoRNA genes are conserved and their changes in the genome are more traceable, which are extremely suited for studying mechanisms underlying intron-exon loss and gain during evolution. Through this study, we revealed that introns could be movable elements in the genome to propel evolution, and hence intron-containing sequences are more prone to sustainable variations leading to evolution.

### Materials

#### Fungal species for intron loss and gain analysis

As referenced by molecular systematic studies [24], [34], [35], we chose different fungal species in our intron loss and gain analysis: *Saccharomyces cerevisiae, Saccharomyces paradoxus, Saccharomyces bayanus, Candida glabrata, Saccharomyces castellii, Saccharomyces kluyveri, Kluyveromyces waltii, Ashbya gossypii, Pichia guilliermondii, Debaryomyces hansenii, Clavispora lusitaniae, Lodderomyces elongisporus, Candida parapsilosis, Candida tropicalis, Candida dubliniensis, Candida albicans* and *Yarrowia lipolytica* belong to the basal species in *Hemiascomycetes. Podospora anserine, Chaetomium globosum, Neurospora crassa, Magnaporthe grisea, Gibberella zeae, Gibberella moniliformis, Phaeosphaeria nodorum, Aspergillus flavus, Coccidioides immitis, Sclerotinia sclerotiorum, Aspergillus fumigatus, Neosartorya fischeri, Aspergillus terreus* and *Aspergillus nidulans* are the basal species in *Euascomycetes*. *Schizosaccharomyces japonicus, Schizosaccharomyces pombe, Schizosaccharomyces octosporus* and *Schizosaccharomyces cryophilus* belong to *Archiascomycetes* and *Coprinopsis cinerea, Phanerochaete chrysosporium* and *Ustilago maydis* are basal species of *Basidiomycota*. *Rhizopus oryzae* belongs to *Zygomycota*.

#### Survey of snoRNA gene cluster I, II and III sequences in fungi

To obtain sequences of snoRNA gene cluster I, II and III from above fungal species, we downloaded budding yeast box C/D snoRNA sequences from the snoRNA database (http://people.biochem.umass.edu/fournierlab/snornadb) and used the sequences of *S. cerevisiae* snoRNA cluster I, II and III sequences as query to search for their orthologs in other fungi from multiple complete genome sequences as well as high-quality draft sequences by the BLAST tool on the NCBI website (http://www.ncbi.nlm.nih.gov/sutils/genom_tree.cgi).

#### Prediction of snoRNA-associated introns

The intron sequences in *Y. lipolytica, D. hansenii, S. cerevisiae, C. glabrata* and *K. lactis* were downloaded from the Génosplicing website (http://genome.jouy.inra.fr/genosplicing/index), and the splicing pattern of these organisms were analyzed. In addition, introns and splicing elements of five diverse Fungi, two filamentous members of the *Ascomycota*, *A. nidulans* and *N. crassa*, a member of the *Basidiomycota*, *Cryptococcus neoformans*, and two well-studied members of the *Ascomycota* group of fungal organisms, *S. cerevisiae* and *S. pombe* were compared and analyzed [30]. From the analyses we accurately characterized conserved fungal intronic elements and predicted snoRNA-associated introns in snoRNA gene cluster I, II and III.

#### Splicing analysis of snoRNA-associated introns

The availability of genomic sequences and expressed sequence tag (EST) data of some fungi permitted the identification of intron for these organisms by aligning ESTs to genomic sequences. snoRNA-associated introns in cluster I, II and III from some fungi were confirmed by the comparison of EST data from these species to the corresponding genomic sequences. In addition, the splicing of snoRNA-associated introns in cluster I and II of *C. albicans* [15] and in cluster I, II and III of *S. pombe* were confirmed previously.

Fungal species *D. hansenii*, *Y. lipolytica, N. crassa, C. glabrata* and *K. lactis* were used for the experimental confirmation of intron structure and splicing pattern. These strains were grown in rich YPD medium (1% yeast extract, 1% peptone, 2% glucose) at 30°C. *Escherichia coli* strain TG1 [F’/supE, hsd△5, thi△(lac-proAB)] grown in 2YT(1.6% Bacto tryptone, 1% Bacto yeast extract, 0.5% NaCl) liquid or solid medium and were used for cloning procedures. Total RNA was extracted from cells grown on YPD medium with the use of guanidine thiocyanate/phenol-chloroform extraction. Reverse transcription was carried out in 20 µL reaction mixture containing 15 µg of total cellular RNA treated with DNase, reverse primer and 500 mmol/L dNTPs. After being denatured at 65°C for 5 min and then cooled to 42°C, 200 units of M-MLV reverse transcriptase (Promega) were added and the extension was carried out at 42°C for 45 min. After reverse transcription, PCR was carried out. The primer pairs DhC2F1/DhC2R1 and DhC2F2/DhC2R1 were used for PCR amplification of *D. hansenii* cluster II. The primer pairs YlC2F1/YlC2R1 and YlC2F2/YlC2R1 were used for PCR amplification of *Y. lipolytica* cluster II. The primer pair DhC3F1/DhC3R1 was used for PCR amplification of *D. hansenii* cluster III. The primer pair NcC2F1/NcC2R1 was used for PCR amplification of *N. crassa* cluster II. The primer pair CgC2F1/CgC2R1 was used for PCR amplification of *C. glabrata* cluster II. The primer pair KlC2F1/KlC2R1 was used for PCR amplification of *K. lactis* cluster II.

DhC2F1: 5’-ACCTAAACTCTACTATAATG-3’

DhC2F2: 5’-GAAGTATTGGTATGTTTCAAC-3’

DhC2R1: 5’- GAGTTCTGAAGTATATTAAG-3’

YlC2F1: 5’- CTCACATACGACAAGACAATG-3’

YlC2F2: 5’-CACGACACTGAATGGTGAGTAC-3’

YlC2R1: 5’- TACGTTAGCTATAAATCAGGG-3’

DhC3F1: 5’- ATATATGGAATCACTGAAAG-3’

DhC3R1: 5’- CATGTATTCATAAGAATTGG-3’

NcC2F: 5’- TGGTTCGCACGGATAGA-3’

NcC2R: 5’- CCCACTAGACGCAAGAT-3’

CgC2F: 5’- AATTTTTTCAATGCTAATGGT-3’

CgC2R: 5’- AACGTATCTCCCCGTTTTCAA-3’

KlC2F: 5’- CTACCGATTCTAAATGATTAT-3’

KlC2R: 5’- GCCTTTCTATATTTCAAGTAT-3’

The RT-PCR amplified fragments were cloned into plasmid vectors to construct cDNA libraries. Then the clones from these libraries were sequenced to confirm the splicing snoRNA-associated introns.

### Phylogenetic analysis

Sequences of the surrounding exons of snR51-associated nested introns in *Euascomycetes* snR41-snR70-snR51 polycistronic cluster III were got and aligned in Clustal X. Alignments without introns were used to build gene tree with neighbor joining and calculation of bootstrap values with MEGA 4.

## Supporting Information

Figure S1 **Splicing of cluster II introns in** ***Yarrowia lipolytica*** **.** (*A*) snoRNA cluster II DNA sequence from *Y. lipolytica*. Coding regions for snoRNAs are underlined. The exons of the non-coding RNA are in capital letters. Introns are in lowercase letters. Conserved 5’splice canonical sequences are in blue. Branch-point sequences and the 3’splice canonical sequences are in red. Arrows mark the locations of the primers used for RT-PCR analysis. (*B*) Partial sequence of splice intermediate. Arrow indicates position where the first intron is removed. (*C*) Partial sequence of spliced end product. Arrow in red indicates position where the first and second introns are removed. Arrow in black indicates position where the third intron is removed. (*D*) Schematic diagram of the structure and expression of snoRNA gene cluster II from *Y lipolytica*.
(TIF)

Figure S2 **Splicing of cluster II introns in** ***Debaryomyces hansenii*** **.** (*A*) snoRNA cluster II DNA sequence from *D. hansenii*. Coding regions for snoRNAs are underlined. The exons of the non-coding RNA are in capital letters. Introns are in lowercase letters. Conserved 5’splice canonical sequences are in blue. Branch-point sequences and the 3’splice canonical sequences are in red. Shared nucleotide by two introns is in green and marked by red asterisk. Arrows mark the locations of the primers used for RT-PCR analysis. (*B*) Partial sequence of splice intermediate. Arrow indicates position where the intron I is removed. (*C*) Partial sequence of spliced end product. Arrow in red indicates position where intron I and II are removed. Arrow in black indicates position where intron III is removed. (*D*) Schematic diagram of the structure and expression of snoRNA gene cluster II from *D. hansenii*.
(TIF)

Figure S3 **Splicing of cluster III introns from** ***Debaryomyces hansenii***. (*A*) snoRNA cluster III DNA sequence from *D. hansenii*. Coding regions for snoRNAs are underlined. The exons of the non-coding RNA are in capital letters. Introns are in lowercase letters. Conserved 5’splice canonical sequences are in blue. Branch-point sequences and the 3’splice canonical sequences are in red. Arrows mark the locations of the primers used for RT-PCR analysis. (*B*) Partial sequence of splice intermediate. Arrow indicates position where the first intron is removed. (*C*) Partial sequence of spliced end product. Arrow indicates position where the first and second introns are removed. (*D*) Schematic diagram of the structure and expression of snoRNA gene cluster III from *D. hansenii*.
(TIF)

Figure S4 **Junction sequences of recently gained introns.** (*A*) Junction sequences of recently gained snoRNA-associated intron in fungi. (*B*) Junction sequences of recently gained non snoRNA-associated intron in fungi. (*C*) Junction sequences of recently gained intron in protein coding gene of *Daphnia*. Intronic sequences are set in lowercase letters and flanking exon sequences in capital letters. The short inverted repeats sequences and short direct repeats sequences are indicated with black arrow and blue arrow, respectively.
(TIF)

Figure S5 **Sequence alignment showing intron gains in** ***Euascomycetes***. Conserved intronic sequences are set in gray and flanking exon sequences in multicolor.
(TIF)

Figure S6 Neighbor-joining gene tree of the surrounding exon sequences of snR51-associated nested introns in *Euascomycetes* snR41-snR70-snR51 polycistronic cluster III.
(TIF)

Figure S7 **Comparison of expressed sequence tag (EST) from** ***Euascomycetes*** **species to their corresponding genome sequences.** Coding regions for snoRNAs are in gray, the exons of the non-coding RNA are in capital letters; introns are in lowercase letters; Conserved 5’splice canonical sequences are in blue, branch-point sequences and the 3’splice canonical sequences are in red. Arrows indicates position where the intron is removed.
(TIF)

Figure S8 **Comparison of expressed sequence tag (EST) from** ***Euascomycetes*** **species to their corresponding genome sequences.** Coding regions for snoRNAs are in gray, the exons of the non-coding RNA are in capital letters; introns are in lowercase letters; Conserved 5’splice canonical sequences are in blue, branch-point sequences and the 3’splice canonical sequences are in red. Arrows indicates position where the intron is removed.
(TIF)

Figure S9 **Comparison of expressed sequence tag (EST) from** ***Euascomycetes*** **species to their corresponding genome sequences.** Coding regions for snoRNAs are in gray, the exons of the non-coding RNA are in capital letters; introns are in lowercase letters; Conserved 5’splice canonical sequences are in blue, branch-point sequences and the 3’splice canonical sequences are in red. Arrows indicates position where the intron is removed.
(TIF)

Figure S10 **Comparison of expressed sequence tag (EST) from** ***Euascomycetes*** **species to their corresponding genome sequences.** Coding regions for snoRNAs are in gray, the exons of the non-coding RNA are in capital letters; introns are in lowercase letters; Conserved 5’splice canonical sequences are in blue, branch-point sequences and the 3’splice canonical sequences are in red. Arrows indicates position where the intron is removed.
(TIF)

Figure S11 **Comparison of expressed sequence tag (EST) from** ***Euascomycetes*** **species to their corresponding genome sequences.** Coding regions for snoRNAs are in gray, the exons of the non-coding RNA are in capital letters; introns are in lowercase letters; Conserved 5’splice canonical sequences are in blue, branch-point sequences and the 3’splice canonical sequences are in red. Arrows indicates position where the intron is removed.
(TIF)

Figure S12 **Comparison of expressed sequence tag (EST) from** ***Euascomycetes*** **species to their corresponding genome sequences.** Coding regions for snoRNAs are in gray, the exons of the non-coding RNA are in capital letters; introns are in lowercase letters; Conserved 5’splice canonical sequences are in blue, branch-point sequences and the 3’splice canonical sequences are in red. Arrows indicates position where the intron is removed.
(TIF)

Figure S13 **Comparison of expressed sequence tag (EST) from** ***Euascomycetes*** **species to their corresponding genome sequences.** Coding regions for snoRNAs are in gray, the exons of the non-coding RNA are in capital letters; introns are in lowercase letters; Conserved 5’splice canonical sequences are in blue, branch-point sequences and the 3’splice canonical sequences are in red. Arrows indicates position where the intron is removed.
(TIF)

Figure S14 **Comparison of expressed sequence tag (EST) from** ***Euascomycetes*** **species to their corresponding genome sequences.** Coding regions for snoRNAs are in gray, the exons of the non-coding RNA are in capital letters; introns are in lowercase letters; Conserved 5’splice canonical sequences are in blue, branch-point sequences and the 3’splice canonical sequences are in red. Arrows indicates position where the intron is removed.
(TIF)

Table S1 **Certificated snoRNA-associated introns. Intronic snoRNA sequences are in gray, C and D boxes are indicated and functional sequences are in red.**
(DOC)

Table S2 **snoRNA-associated introns and adjacent exons. Intronic sequences are set in lowercase letters and flanking exon sequences in capital letters.** snoRNA sequences are in gray and short direct repeats sequences are in red.
(DOC)

Table S3 **Exon loss in fungi. Intronic sequences are set in lowercase letters and flanking exon sequences in capital letters.** Conserved 5’splice canonical sequences are in blue, branch-point sequences and the 3’splice canonical sequences are in red.
(DOC)
