# Supplementary figures and images for: Splicing-Related Features of Introns Serve to Propel Evolution
Source: PLoS One. 2013 Mar 13;8(3):e58547. doi: 10.1371/journal.pone.0058547 (PMC3596301; doi:10.1371/journal.pone.0058547)

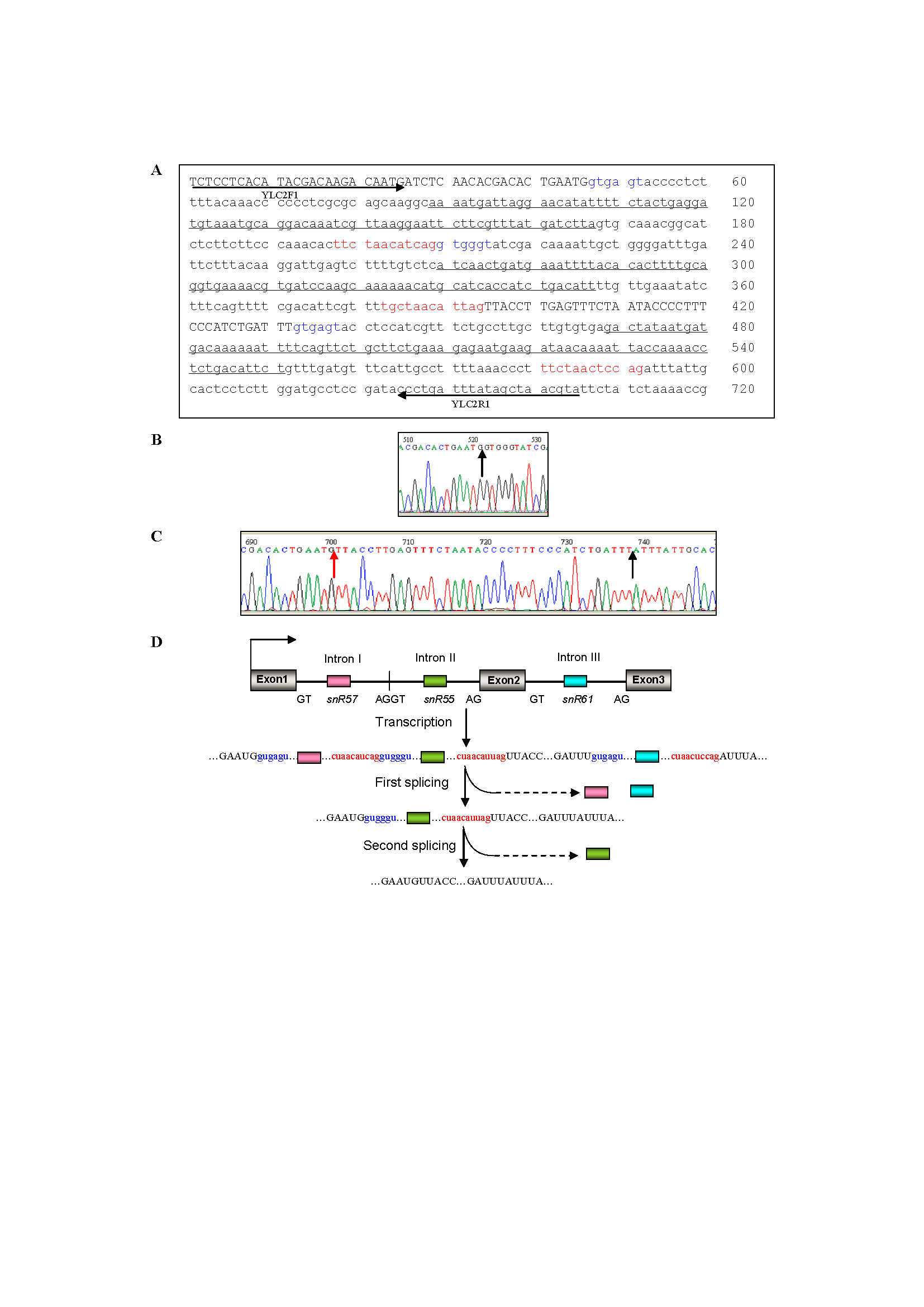

Supplement: Figure S1 — Splicing of cluster II introns in Yarrowia lipolytica . (A) snoRNA cluster II DNA sequence from Y. lipolytica. Coding regions for snoRNAs are underlined. The exons of the non-coding RNA are in capital letters. Introns are in lowercase letters. Conserved 5’splice canonical sequences are in blue. Branch-point sequences and the 3’splice canonical sequences are in red. Arrows mark the locations of the primers used for RT-PCR analysis. (B) Partial sequence of splice intermediate. Arrow indicates position where the first intron is removed. (C) Partial sequence of spliced end product. Arrow in red indicates position where the first and second introns are removed. Arrow in black indicates position where the third intron is removed. (D) Schematic diagram of the structure and expression of snoRNA gene cluster II from Y lipolytica. (TIF) [file pone.0058547.s001.tif]

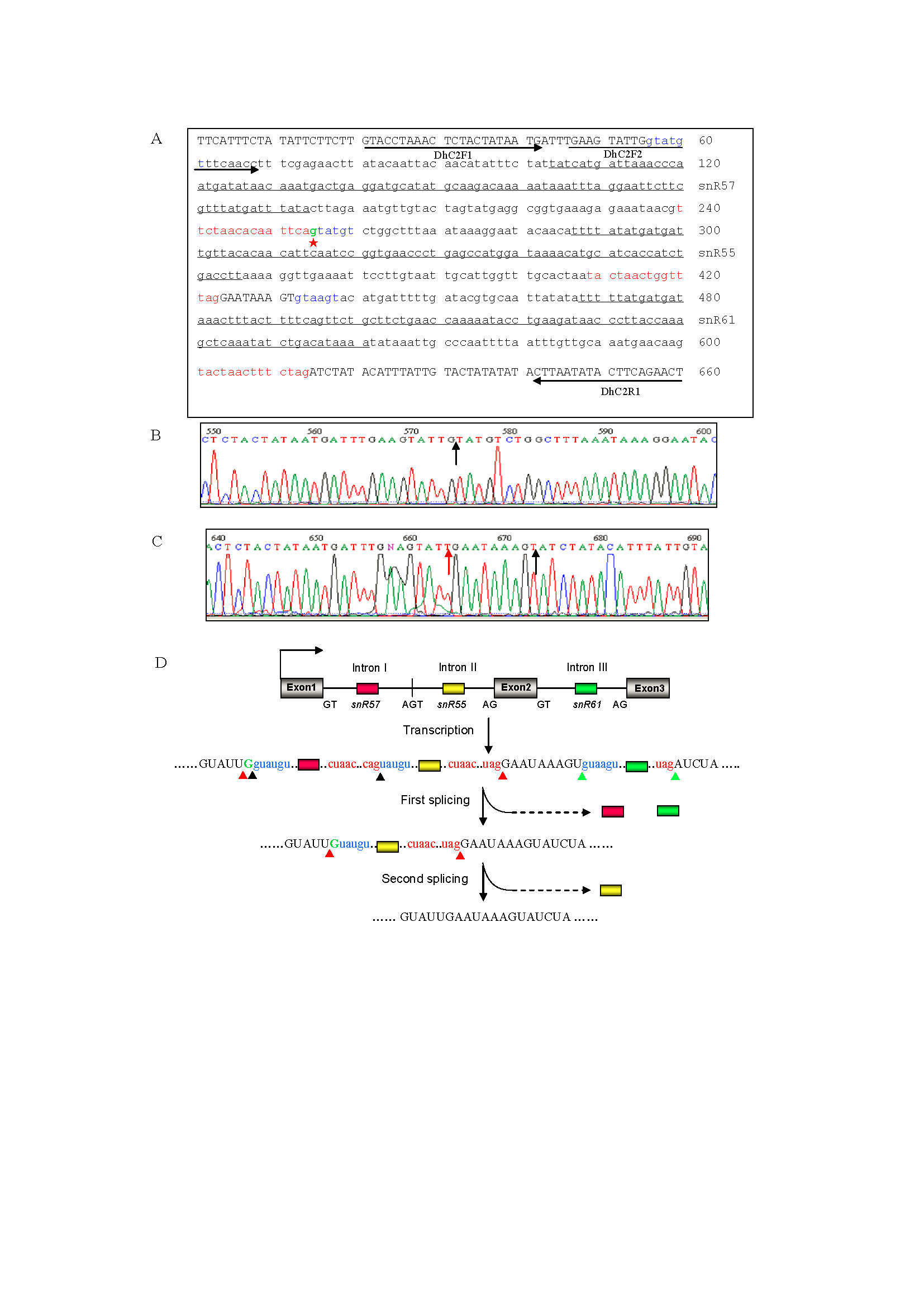

Supplement: Figure S2 — Splicing of cluster II introns in Debaryomyces hansenii . (A) snoRNA cluster II DNA sequence from D. hansenii. Coding regions for snoRNAs are underlined. The exons of the non-coding RNA are in capital letters. Introns are in lowercase letters. Conserved 5’splice canonical sequences are in blue. Branch-point sequences and the 3’splice canonical sequences are in red. Shared nucleotide by two introns is in green and marked by red asterisk. Arrows mark the locations of the primers used for RT-PCR analysis. (B) Partial sequence of splice intermediate. Arrow indicates position where the intron I is removed. (C) Partial sequence of spliced end product. Arrow in red indicates position where intron I and II are removed. Arrow in black indicates position where intron III is removed. (D) Schematic diagram of the structure and expression of snoRNA gene cluster II from D. hansenii. (TIF) [file pone.0058547.s002.tif]

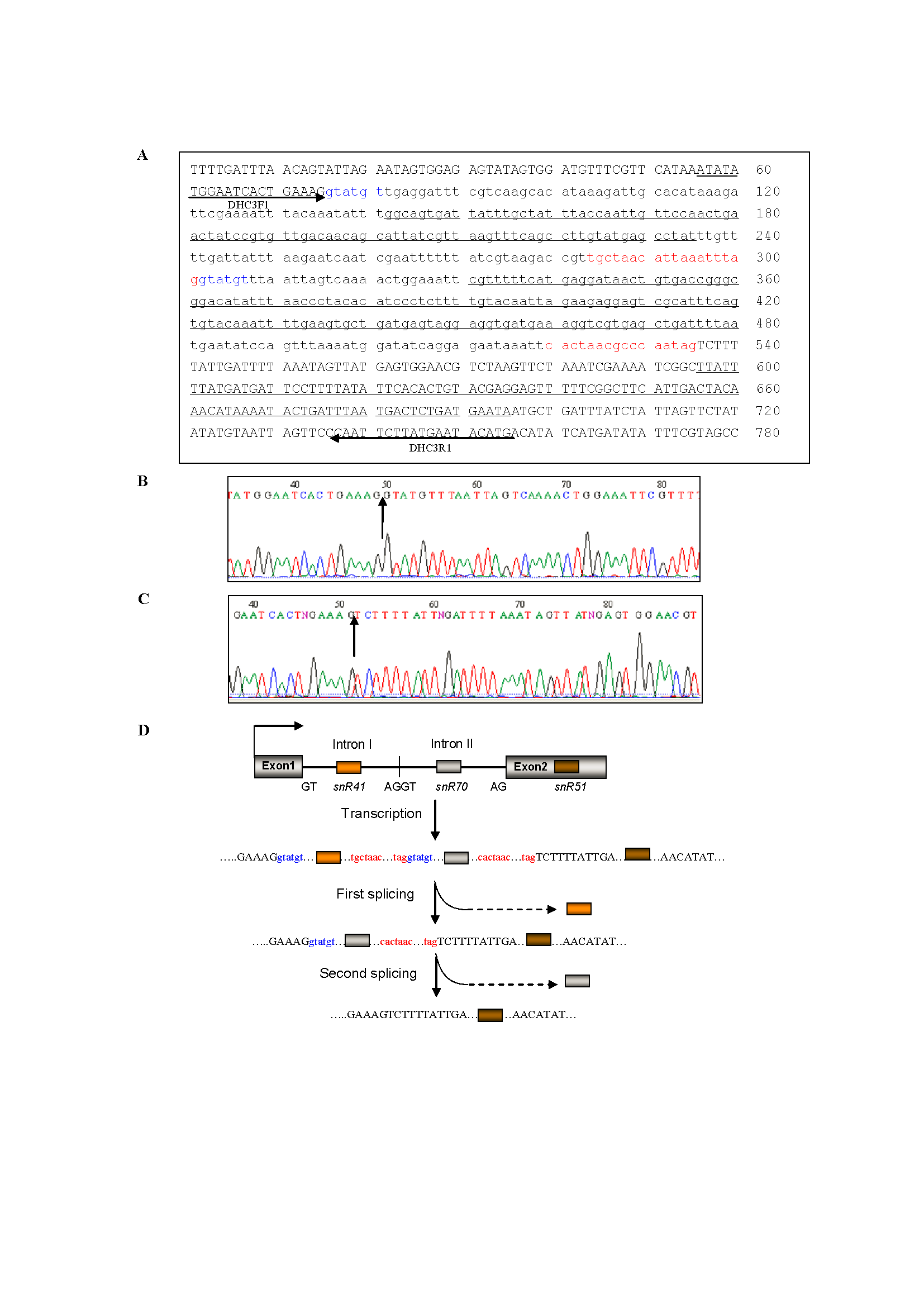

Supplement: Figure S3 — Splicing of cluster III introns from Debaryomyces hansenii. (A) snoRNA cluster III DNA sequence from D. hansenii. Coding regions for snoRNAs are underlined. The exons of the non-coding RNA are in capital letters. Introns are in lowercase letters. Conserved 5’splice canonical sequences are in blue. Branch-point sequences and the 3’splice canonical sequences are in red. Arrows mark the locations of the primers used for RT-PCR analysis. (B) Partial sequence of splice intermediate. Arrow indicates position where the first intron is removed. (C) Partial sequence of spliced end product. Arrow indicates position where the first and second introns are removed. (D) Schematic diagram of the structure and expression of snoRNA gene cluster III from D. hansenii. (TIF) [file pone.0058547.s003.tif]

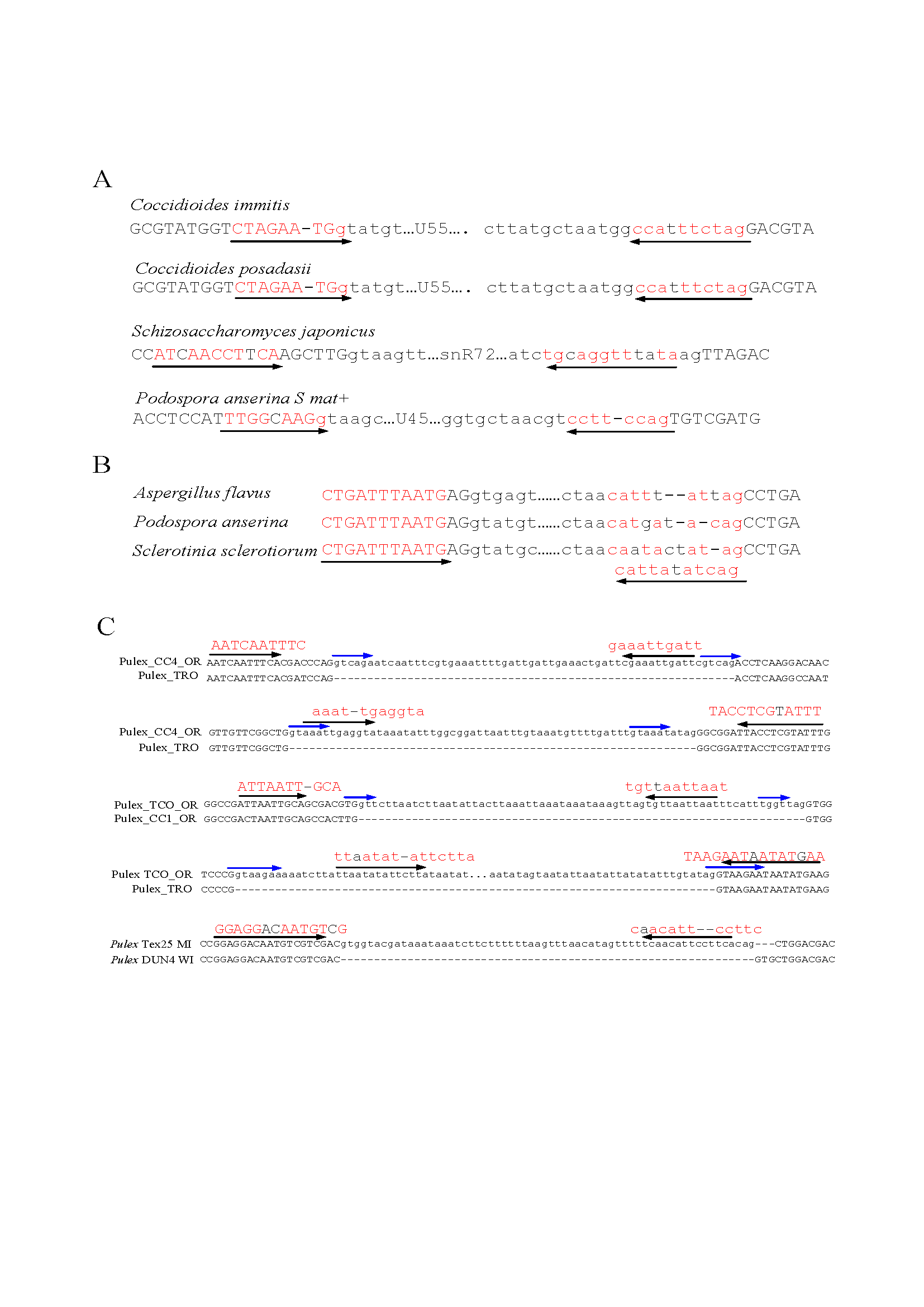

Supplement: Figure S4 — Junction sequences of recently gained introns. (A) Junction sequences of recently gained snoRNA-associated intron in fungi. (B) Junction sequences of recently gained non snoRNA-associated intron in fungi. (C) Junction sequences of recently gained intron in protein coding gene of Daphnia. Intronic sequences are set in lowercase letters and flanking exon sequences in capital letters. The short inverted repeats sequences and short direct repeats sequences are indicated with black arrow and blue arrow, respectively. (TIF) [file pone.0058547.s004.tif]

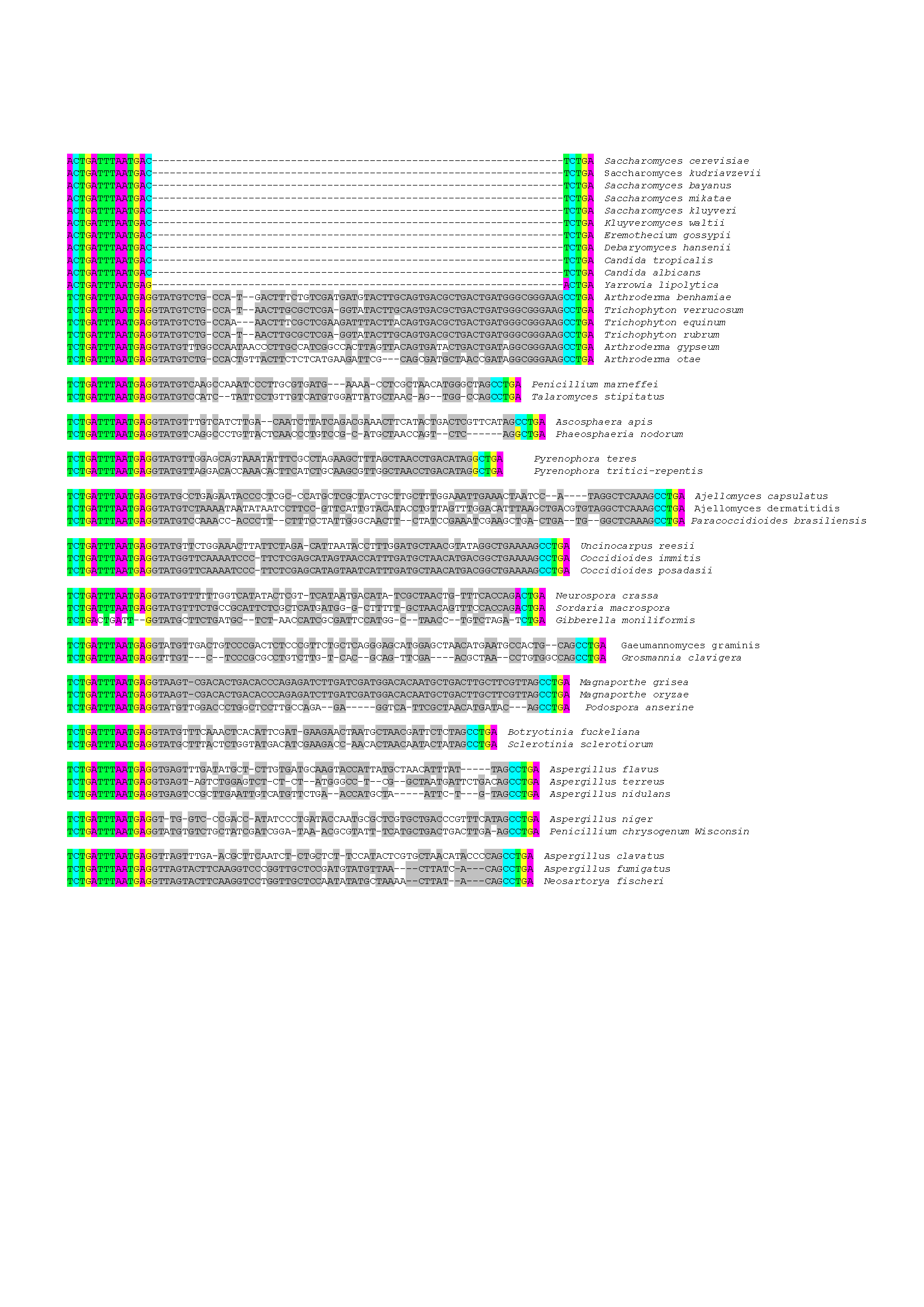

Supplement: Figure S5 — Sequence alignment showing intron gains in Euascomycetes. Conserved intronic sequences are set in gray and flanking exon sequences in multicolor. (TIF) [file pone.0058547.s005.tif]

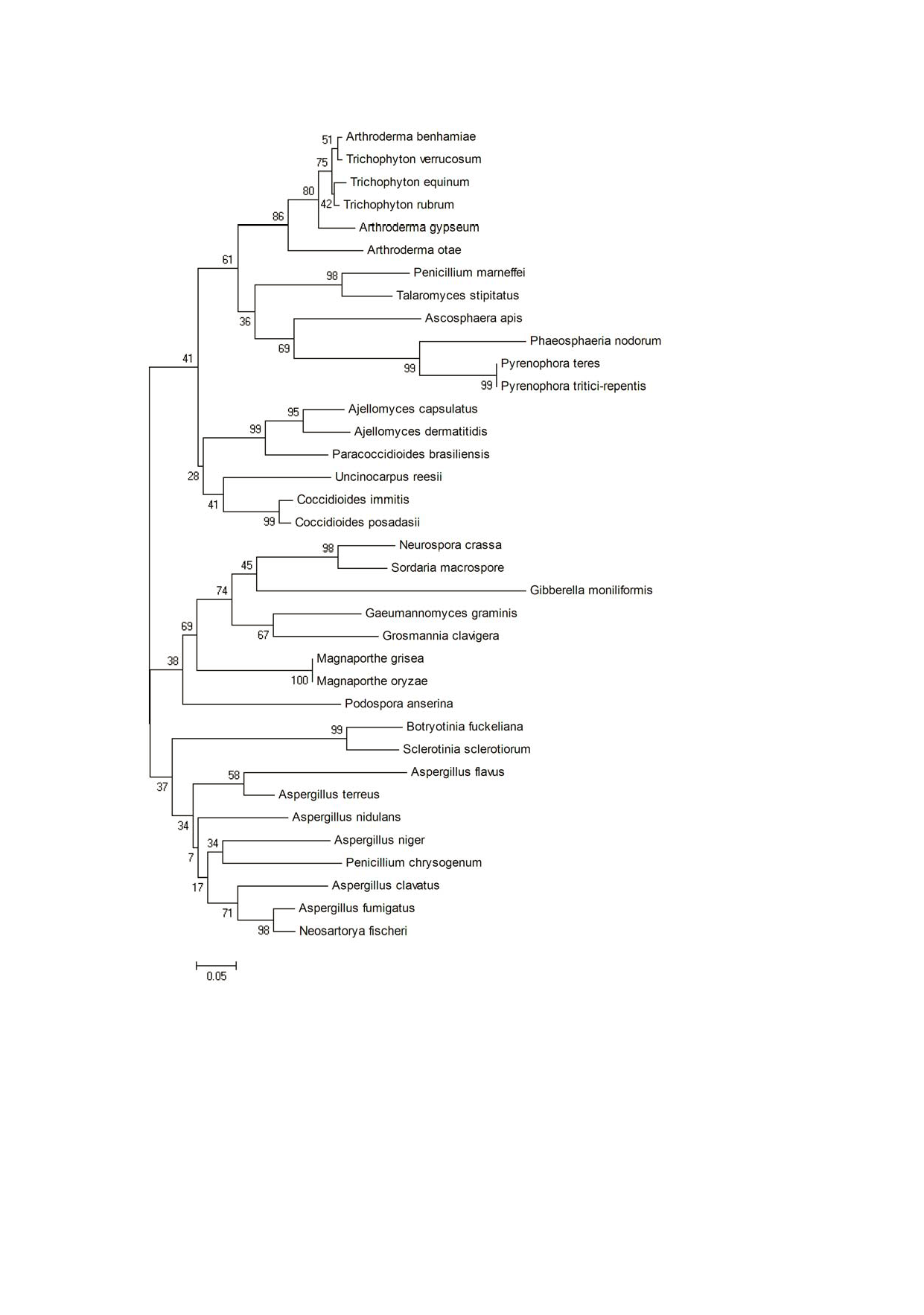

Supplement: Figure S6 — Neighbor-joining gene tree of the surrounding exon sequences of snR51-associated nested introns in Euascomycetes snR41-snR70-snR51 polycistronic cluster III. (TIF) [file pone.0058547.s006.tif]

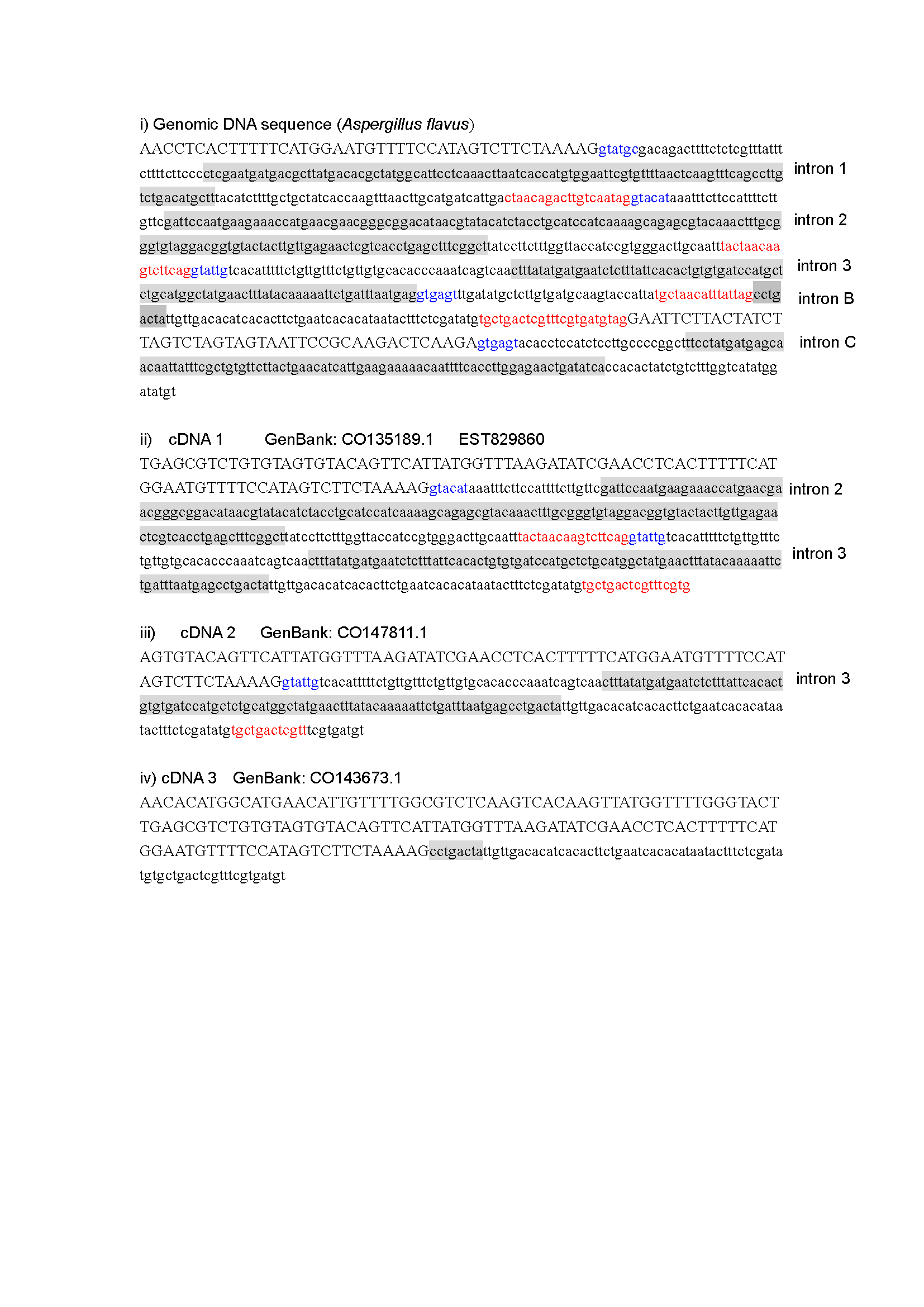

Supplement: Figure S7 — Comparison of expressed sequence tag (EST) from Euascomycetes species to their corresponding genome sequences. Coding regions for snoRNAs are in gray, the exons of the non-coding RNA are in capital letters; introns are in lowercase letters; Conserved 5’splice canonical sequences are in blue, branch-point sequences and the 3’splice canonical sequences are in red. Arrows indicates position where the intron is removed. (TIF) [file pone.0058547.s007.tif]

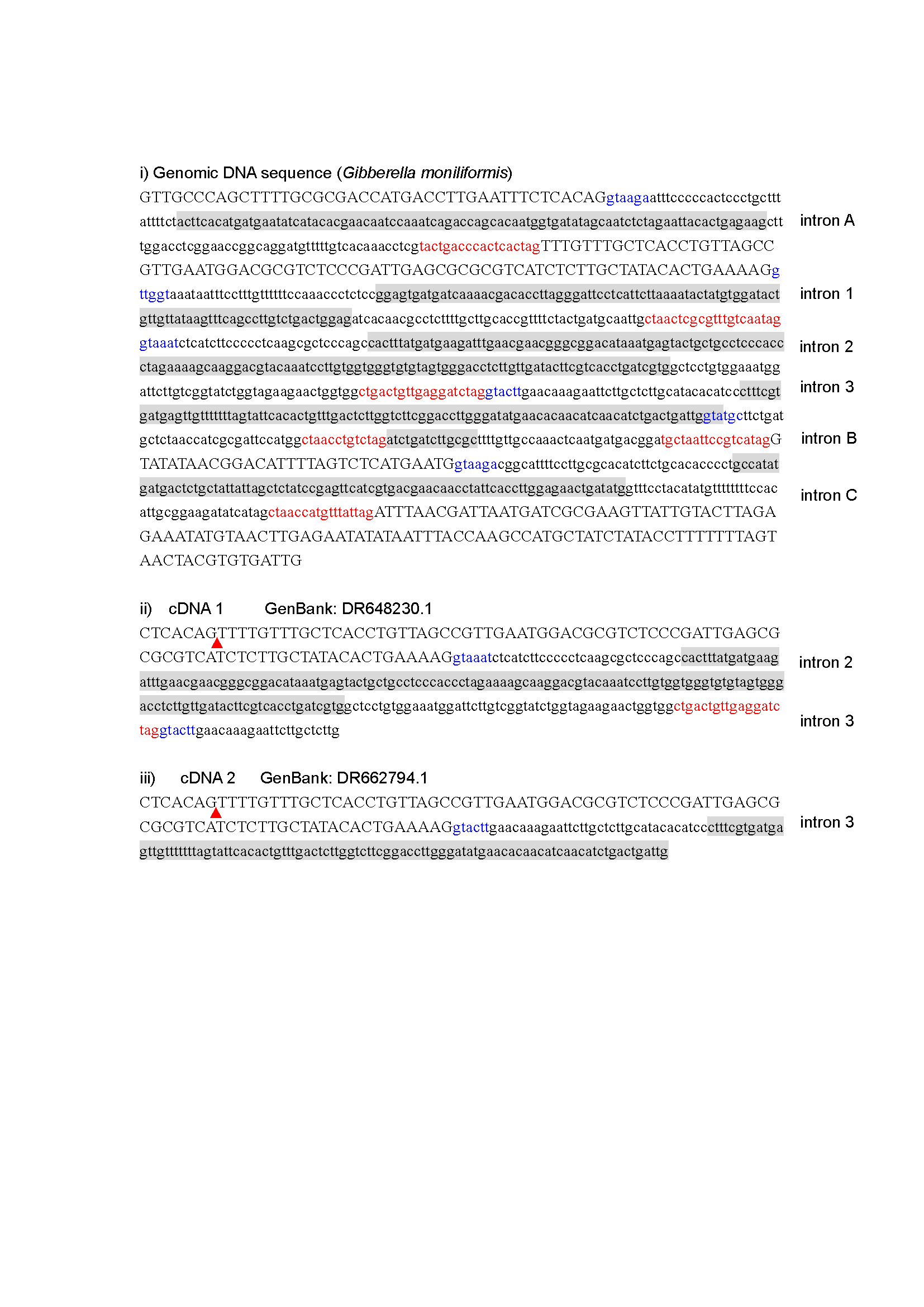

Supplement: Figure S8 — Comparison of expressed sequence tag (EST) from Euascomycetes species to their corresponding genome sequences. Coding regions for snoRNAs are in gray, the exons of the non-coding RNA are in capital letters; introns are in lowercase letters; Conserved 5’splice canonical sequences are in blue, branch-point sequences and the 3’splice canonical sequences are in red. Arrows indicates position where the intron is removed. (TIF) [file pone.0058547.s008.tif]

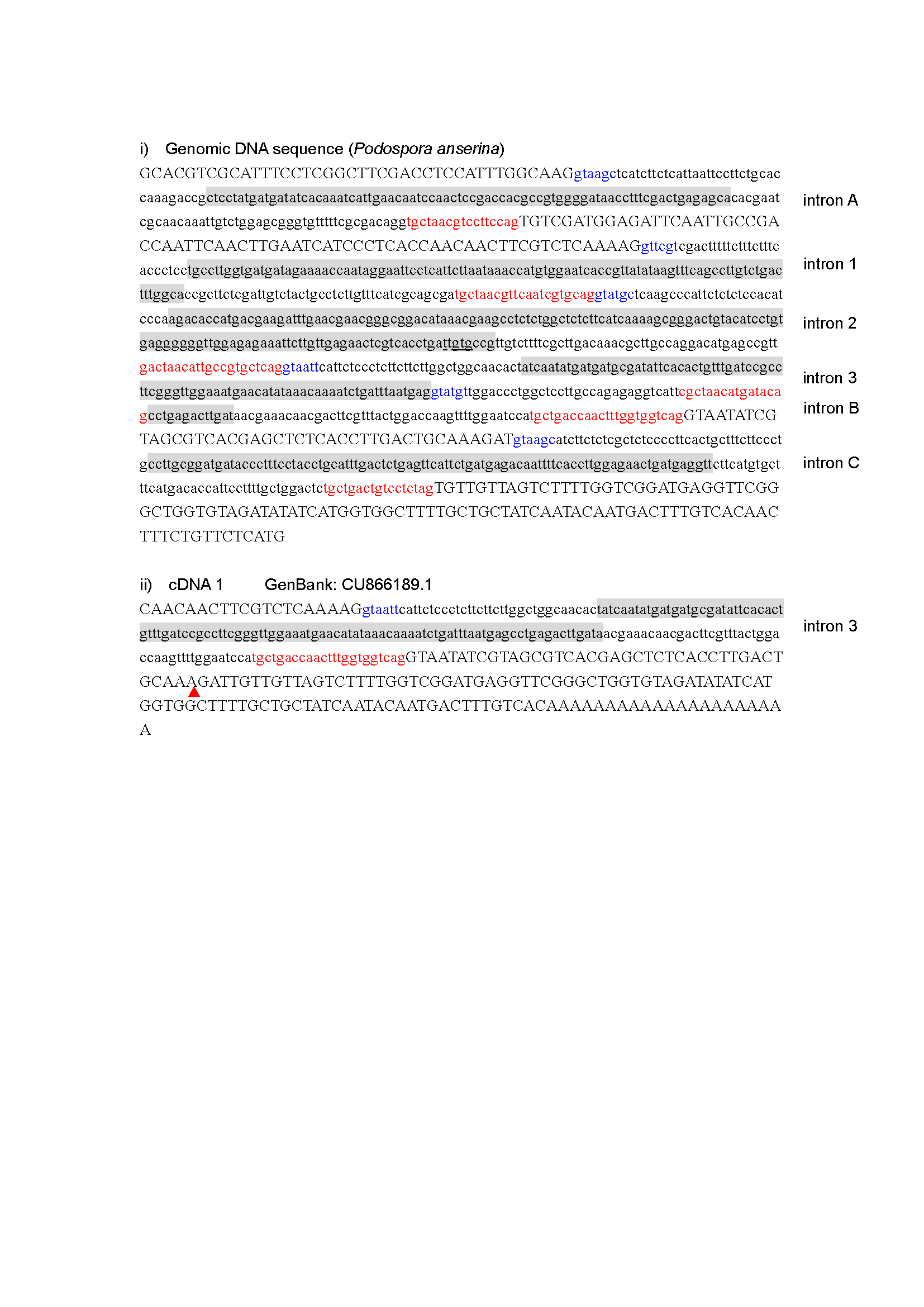

Supplement: Figure S9 — Comparison of expressed sequence tag (EST) from Euascomycetes species to their corresponding genome sequences. Coding regions for snoRNAs are in gray, the exons of the non-coding RNA are in capital letters; introns are in lowercase letters; Conserved 5’splice canonical sequences are in blue, branch-point sequences and the 3’splice canonical sequences are in red. Arrows indicates position where the intron is removed. (TIF) [file pone.0058547.s009.tif]

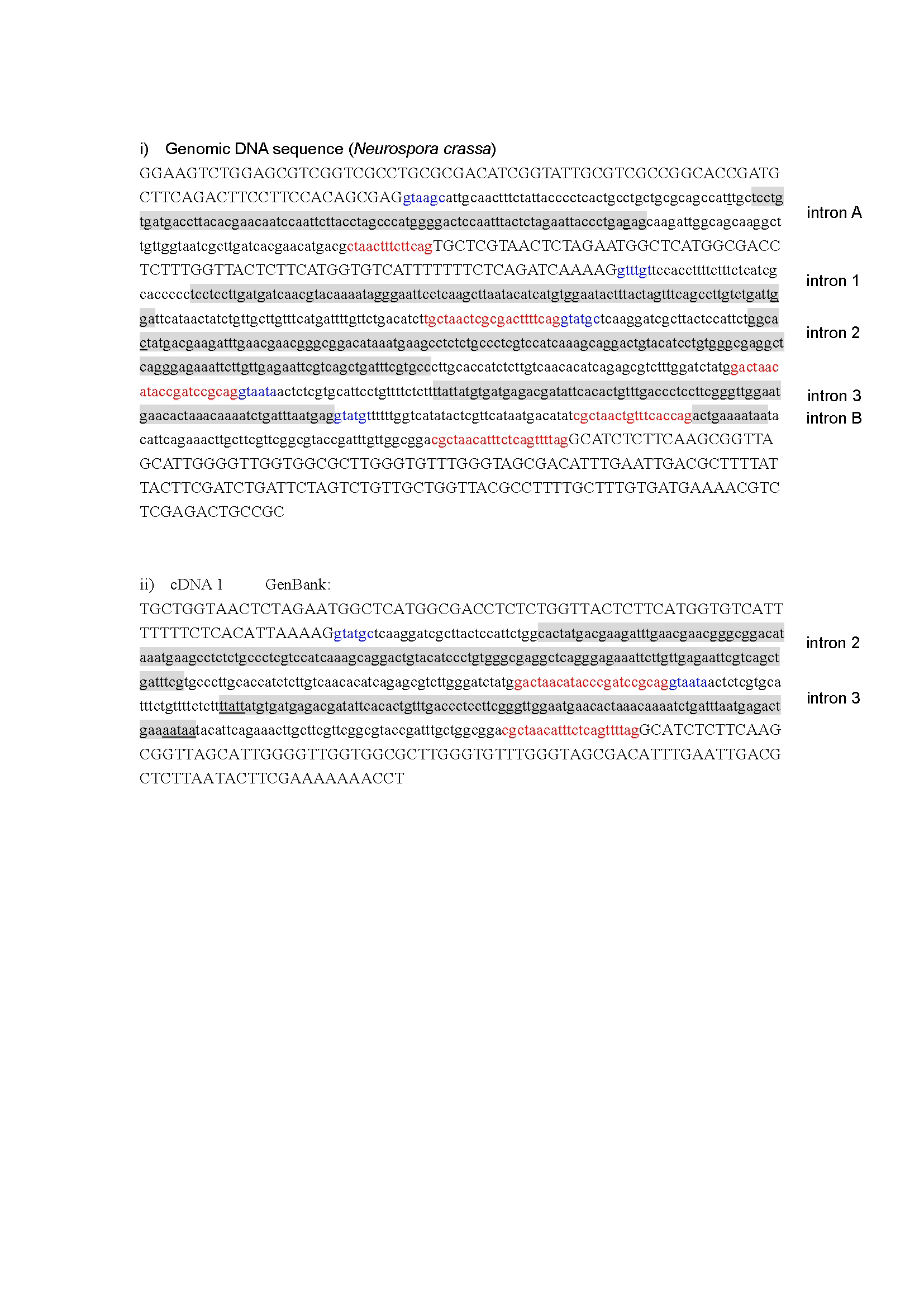

Supplement: Figure S10 — Comparison of expressed sequence tag (EST) from Euascomycetes species to their corresponding genome sequences. Coding regions for snoRNAs are in gray, the exons of the non-coding RNA are in capital letters; introns are in lowercase letters; Conserved 5’splice canonical sequences are in blue, branch-point sequences and the 3’splice canonical sequences are in red. Arrows indicates position where the intron is removed. (TIF) [file pone.0058547.s010.tif]

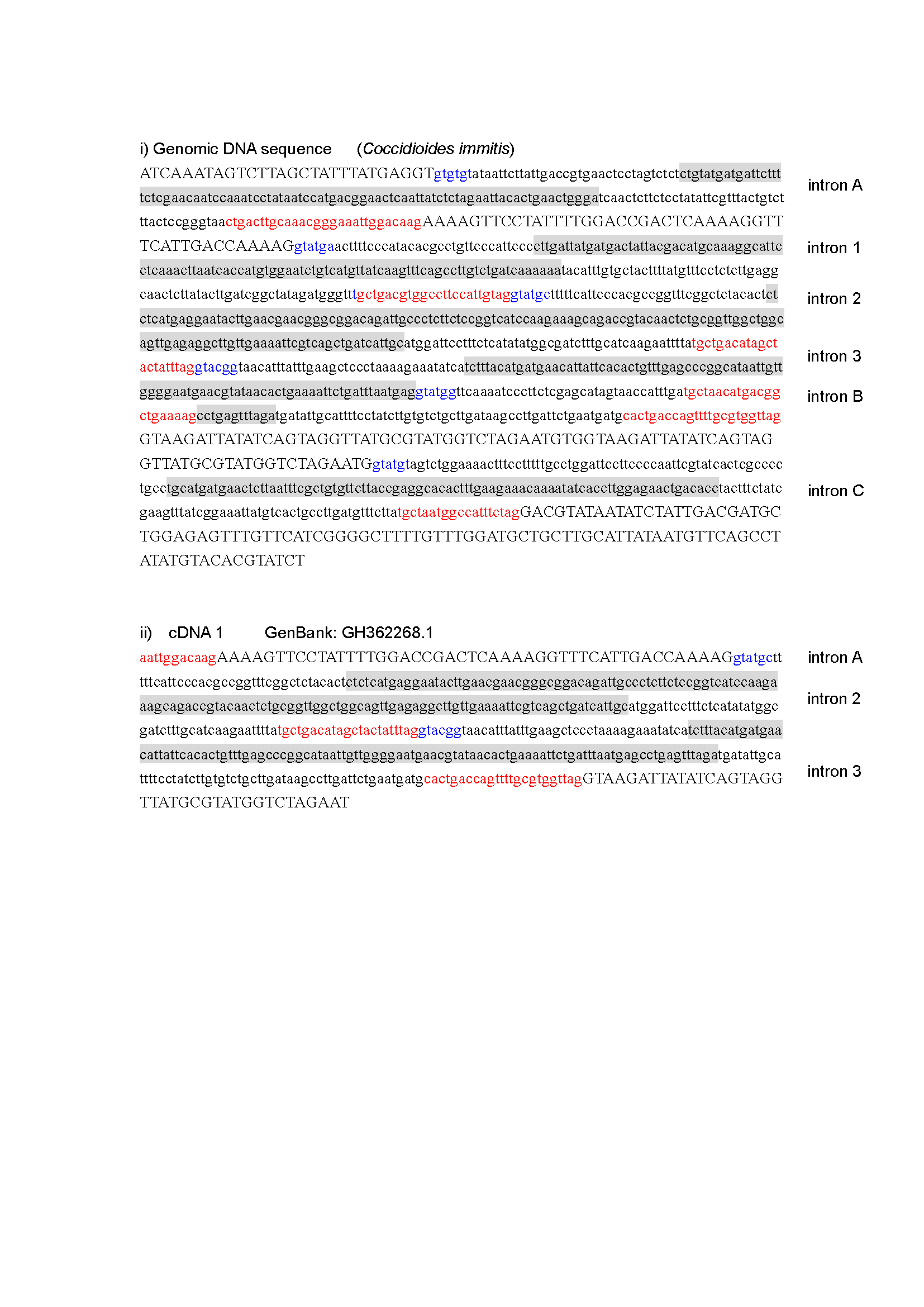

Supplement: Figure S11 — Comparison of expressed sequence tag (EST) from Euascomycetes species to their corresponding genome sequences. Coding regions for snoRNAs are in gray, the exons of the non-coding RNA are in capital letters; introns are in lowercase letters; Conserved 5’splice canonical sequences are in blue, branch-point sequences and the 3’splice canonical sequences are in red. Arrows indicates position where the intron is removed. (TIF) [file pone.0058547.s011.tif]

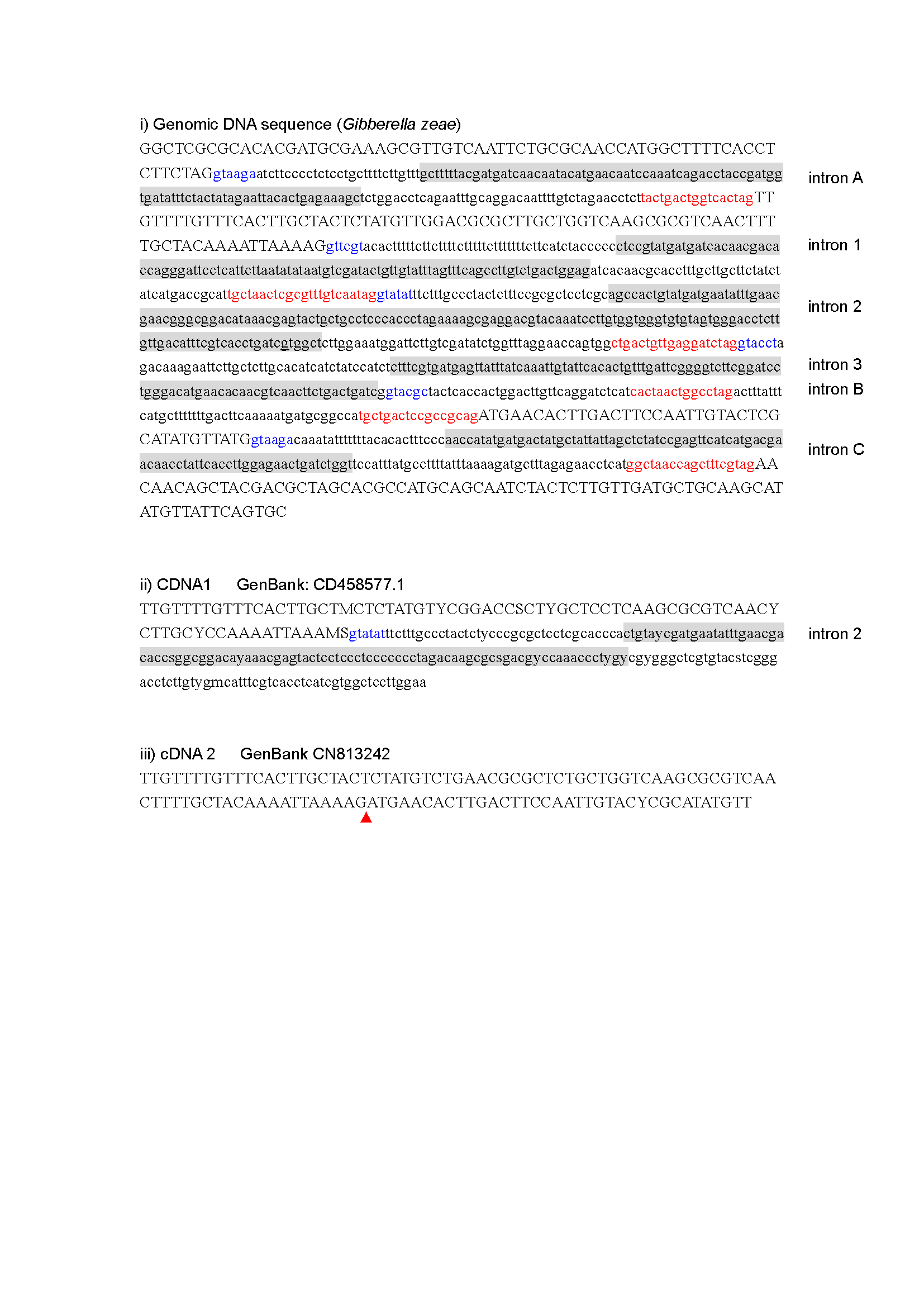

Supplement: Figure S12 — Comparison of expressed sequence tag (EST) from Euascomycetes species to their corresponding genome sequences. Coding regions for snoRNAs are in gray, the exons of the non-coding RNA are in capital letters; introns are in lowercase letters; Conserved 5’splice canonical sequences are in blue, branch-point sequences and the 3’splice canonical sequences are in red. Arrows indicates position where the intron is removed. (TIF) [file pone.0058547.s012.tif]

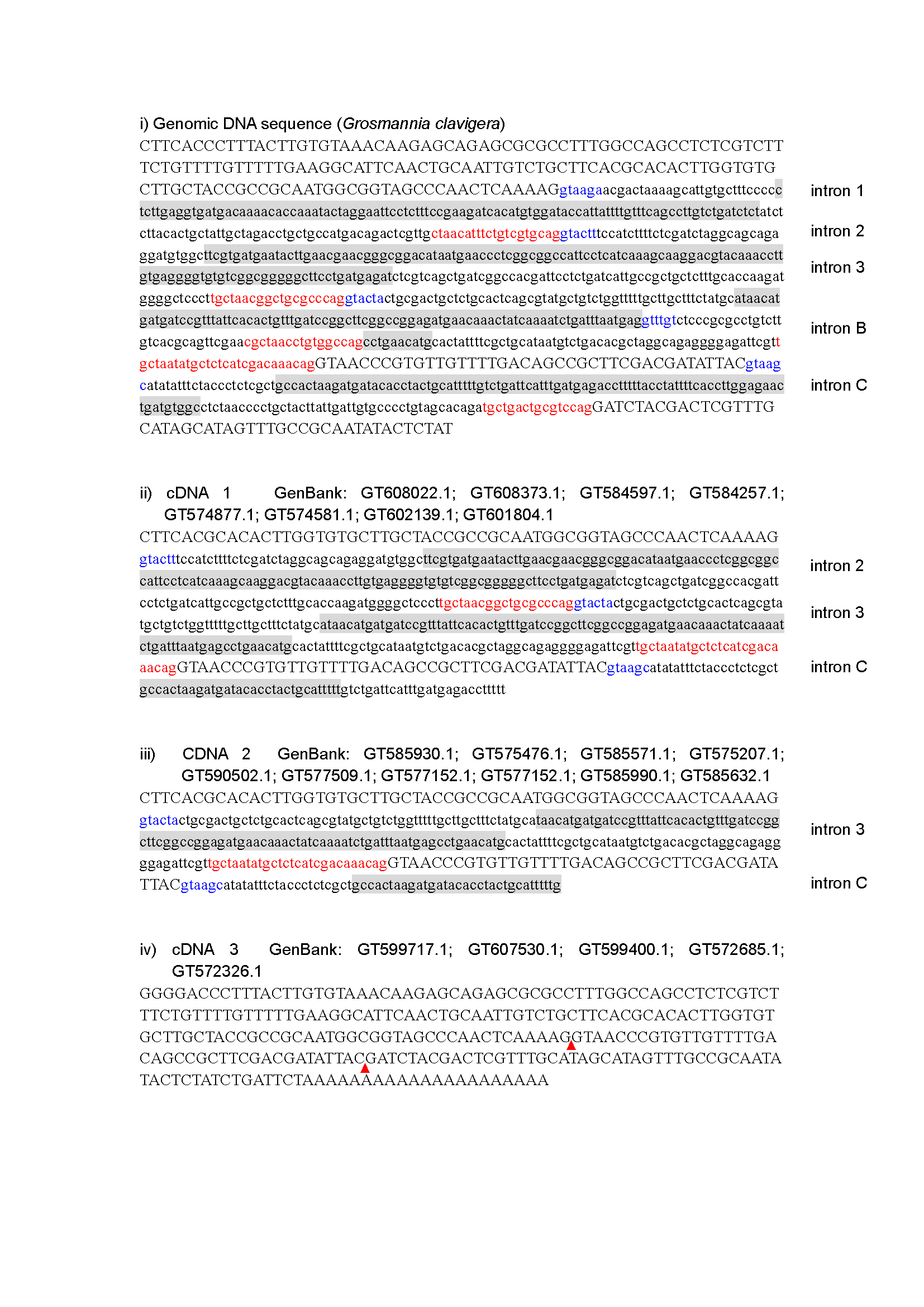

Supplement: Figure S13 — Comparison of expressed sequence tag (EST) from Euascomycetes species to their corresponding genome sequences. Coding regions for snoRNAs are in gray, the exons of the non-coding RNA are in capital letters; introns are in lowercase letters; Conserved 5’splice canonical sequences are in blue, branch-point sequences and the 3’splice canonical sequences are in red. Arrows indicates position where the intron is removed. (TIF) [file pone.0058547.s013.tif]

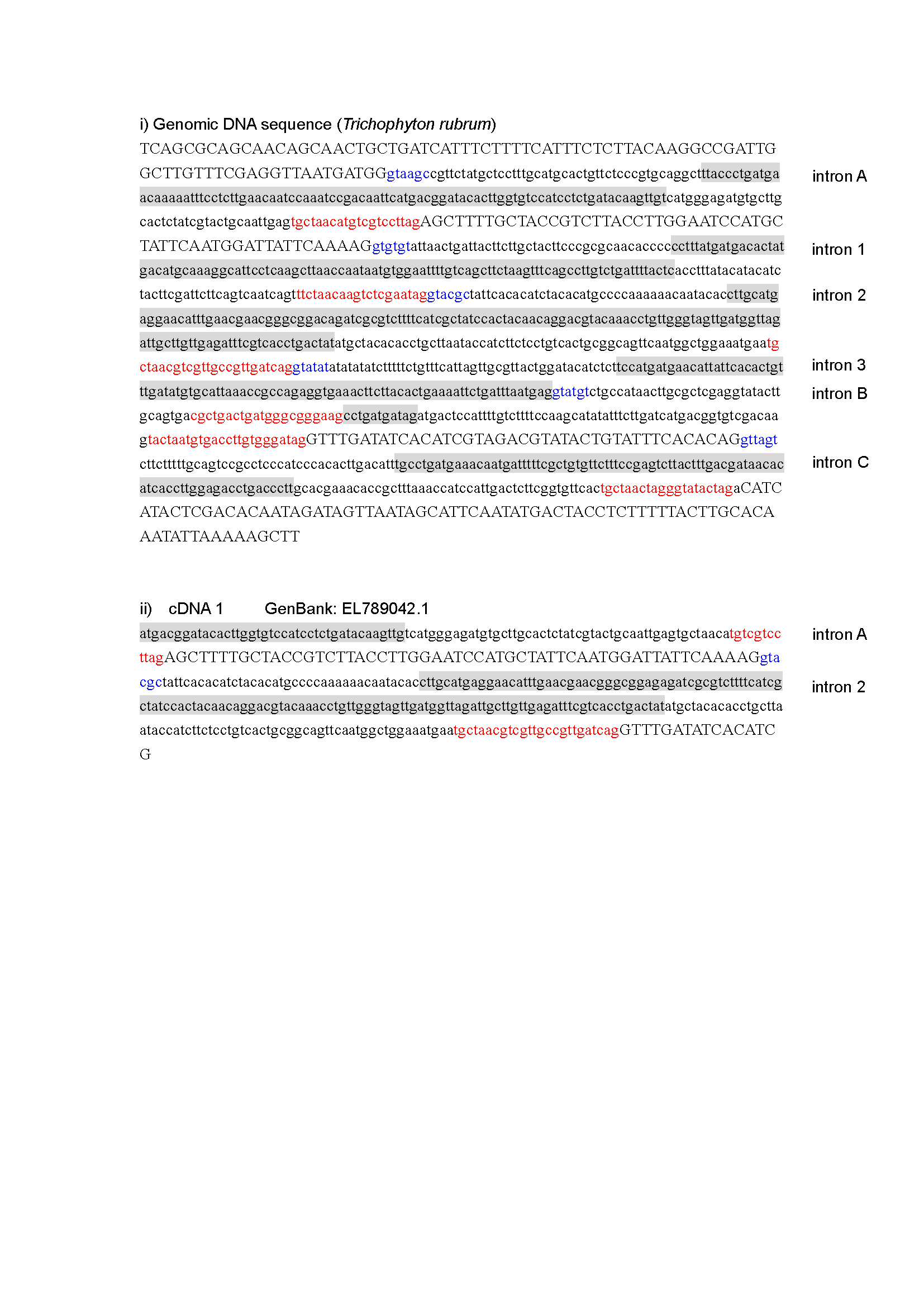

Supplement: Figure S14 — Comparison of expressed sequence tag (EST) from Euascomycetes species to their corresponding genome sequences. Coding regions for snoRNAs are in gray, the exons of the non-coding RNA are in capital letters; introns are in lowercase letters; Conserved 5’splice canonical sequences are in blue, branch-point sequences and the 3’splice canonical sequences are in red. Arrows indicates position where the intron is removed. (TIF) [file pone.0058547.s014.tif]
