# Supplementary material for: Splicing-Related Features of Introns Serve to Propel Evolution
Source: PLoS One. 2013 Mar 13;8(3):e58547. doi: 10.1371/journal.pone.0058547 (PMC3596301; doi:10.1371/journal.pone.0058547)
Supplement: Table S1 — Certificated snoRNA-associated introns. Intronic snoRNA sequences are in gray, C and D boxes are indicated and functional sequences are in red. (DOC) [file pone.0058547.s015.doc]

| **No.** | **species** | **snoRNA** | **snoRNA-associated intron sequences** | **verification** |
| --- | --- | --- | --- | --- |
| 1 | *Debaryomyces hansenii* | snR57 | gtatgtttcaacctttcgagaacttatacaattacaacatatttctattatcatgattaaacccaatgatataacaaatgactgaggatgcatatgcaagacaaaaataaatttaggaattcttcgtttatgatttatacttagaaatgttgtactagtatgaggcggtgaaagagaaataacgttctaacacaattcag | This work |
| 2 | *Debaryomyces hansenii* | snR55 | gtatgtctggctttaaataaaggaatacaacattttatatgatgattgttacacaacattcaatccggtgaaccctgagccatggataaaacatgcatcaccatctgaccttaaaaggttgaaaattccttgtaattgcattggtttgcactaatactaactggtttag | This work |
| 3 | *Debaryomyces hansenii* | snR61 | gtaagtacatgatttttgatacgtgcaattatatatttttatgatgataaactttacttttcagttctgcttctgaaccaaaaatacctgaagataacccttaccaaagctcaaatatctgacataaaatataaattgcccaattttaatttgttgcaaatgaacaagtactaactttctag | This work |
| 4 | *Debaryomyces hansenii* | snR41 | gtatgttgaggatttcgtcaagcacataaagattgcacataaagattcgaaaatttacaaatatttggcagtgattatttgctatttaccaattgttccaactgaactatccgtgttgacaacagcattatcgttaagtttcagccttgtatgagcctatttgttttgattatttaagaatcaatcgaattttttatcgtaagaccgttgctaacattaaatttag | This work |
| 5 | *Debaryomyces hansenii* | snR70 | gtatgtttaattagtcaaaactggaaattcgtttttcatgaggataactgtgaccgggcggacatatttaaccctacacatccctcttttgtacaattagaagaggagtcgcatttcagtgtacaaattttgaagtgctgatgagtaggaggtgatgaaaggtcgtgagctgattttaatgaatatccagtttaaaatggatatcaggagaataaattcactaacgcccaatag | This work |
| 6 | *Candida albicans* | snR78 | gtatgttttaaattaattcttagcaattgaaacattgagattatattcaaaagagaagaaattgaggatgaatacgaataatcatttaaactagagtctgtgacttttaatcgggaatgatcagaaaattattttatgcatggctttctgacaaatttcttttcttcttatacttgtttaatttcaatgttatggaaatacggagaatatatttttactaacttcaatatcag | previous work |
| 7 | *Candida albicans* | snR77 | gtatgttcatcaatattatactttttttctttctttctttcttgctgttgttcaaaaaaaatttttttttatgttttcaatgatgatttaacaaaaacaaagctggaattactggctgaaatctaatgattgataaagtttttgcaccaatacgctcctgatttttttctaaaaaaaaatatttgattaaacaataaataaataaaagagatagagagagaaataacccaattaactatttgaagaagtatatttactaacatttcgattcaag | previous work |
| 8 | *Candida albicans* | snR76 | gtatgtcaataacatataatacatataatatttcttgggggatagtttccttttattgattaggatgaattatcgcactgggcactgaatatatattttatgagtgacaatgcattttaggtgggcctcagaaacccactgtaactgattcatacaaataaactaatccaataattgaatgaataatatttattaggggaataatgtactaacttacatttaaag | previous work |
| 9 | *Candida albicans* | snR75 | gtatgtatttcatatatgggattccccccaaatatctatatatatatttatatgaatatatatatatatgtgaaaaattagatgtgatgattttactagatgacgagtcagaacctcgggtttcccgttgatgacaaacttttatttaattaccattcatgcatttctgatctaagtcaattttattgttgttttatttttttccaaattgaaattgttatactaactaaattaataaag | previous work |
| 10 | *Candida albicans* | snR72 | gtatgtgatacaattttgtttcatattatatatatatatatatatatttattgtgatgatactataccaatttcatctttccattgagtttttccaaaaaaaaaaaagactatgaaaagaacttcactaaaattcgatttgcatgtcagtctgattttcaagaaatatatatatatatatataacatatatggtttcaaattattcataatagaatactaactttcaaaaag | previous work |
| 11 | *Candida albicans* | snR57 | gtatgcaatatagtcacagaaatataatgatgaaatatcatcaaccctggtcttattccatttgttaaacaatatattcaacttgatgattaaaactattgatgtaacattattactgaggatgtacatgcaggataaatagttaaggaattcttcgtttatgaatttagatacattggtttaacaattgggagtatcagagaagattgaatcaatagctgtttactaacaatgtttttttttgaatacag | previous work |
| 12 | *Candida albicans* | snR55 | gtatgtttctcatttgacctttgtattttttgaatgaaaggactgttaatatgatgaatatgtgttaccacacttttctgtccggtgaataaaatatgatccatggatactttacatgcatcaccatctgattataattgactttttcgaaaaaaaaaagtaataaattatatttgtttttcaatactaactgataatttatag | previous work |
| 13 | *Candida albicans* | snR61 | gtatgtataagtgttaacacttttgatttgaaatatattgttatgggttttctattttgatgatgaaaaataaatgttatcagttctgcttctgaaccaaataatctgaagatactcgtttaccaagctctagaattacactgatccaaaatagaaacatccaatatttatttaattcaaatcgatattttgcaatgaataatttaatttcatttatttatttgttgggaaattggaaattagaatcataattactaacaaaatccaattcag | previous work |
| 14 | *Yarrowia lipolytica* | snR57 | gtgagtacccctcttttacaaacccccctcgcgcagcaaggcaaaatgattaggaacatattttctactgaggatgtaaatgcaggacaaatcgttaaggaattcttcgtttatgatcttagtgcaaacggcatctcttcttcccaaacacttctaacatcag | This work |
| 15 | *Yarrowia lipolytica* | snR55 | gtgggtatcgacaaaattgctggggatttgattctttacaaggattgagtcttttgtctcatcaactgatgaaattttacacacttttgcaggtgaaaacgtgatccaagcaaaaaacatgcatcaccatctgacattttgttgaaatatctttcagttttcgacattcgttttgctaacattag | This work |
| 16 | *Yarrowia lipolytica* | snR61 | gtgagtacctccatcgtttctgccttgcttgtgtgagactataatgatgacaaaaaattttcagttctgcttctgaaagagaatgaagataacaaaattaccaaaacctctgacattctgtttgatgtttcattgccttttaaaccctttctaactccag | This work |
| 17 | *Yarrowia lipolytica* | snR41 | gtatgtatcgcacaaaccacagctgtggcgactcaaatgattaagaactttaccaattgttccaaccgacttctgtgaagataaaccggcatcgttaagccattatgaagactctaacgaccgactttgtgtcacacgtttgaaaaccgtttgctaacctca | This work |
| 18 | *Yarrowia lipolytica* | snR70 | gtgagacacagaagatgcggacaaggatttttcaaagatgatttttcgaccgggcggacatacgcggggggcttacataaagcgccacacatgtacagtggtgtgggccttcggtgcttgatgatacggtcttaactgagatttttggatggtaaagccatttctttgagtacgctaacaacag | This work |
| 19 | *Neurospora crassa* | snR55 | gtaagtttatttccccttgcctttcacatctcatgatgatcccataaccacagttctcgcgataatctctgagcgatcgaacaaacatgcactaccatctgactactccttcaacacatccttcacttgcaactcatttggaggaaccgttgactgacaatcaatag | GH026965.1 |
| 20 | *Neurospora crassa* | snR61 | gtaagcatcacattgttttcctttctgttttctttcccccacctgacatcatcacccacccgattgtccatgatgaaacaaaatattttcagttctgcttctgaataataatgagcgataaccaattataccaaaaccttctgatatggcaatcaccccaactctttcttgcaacgacagccctcaccacacatgctgcgagcaagttggctaacaaacttcaag | GH026965.1 |
| 21 | *Neurospora crassa* | snR41 | gtttgttccaccttttctttctcatcgcaccccctcctccttgatgatcaacgtacaaaatagggaattcctcaagcttaatacatcatgtggaatactttactagtttcagccttgtctgattggattcataactatctgttgcttgtttcatgattttgttctgacatcttgctaactcgcgacttttcag | GH002039.1 |
| 22 | *Neurospora crassa* | U45 | gtaagcattgcaactttctattacccctcactgcctgctgcgcagccatttgctcctgtgatgaccttacacgaacaatccaattcttacctagcccatggggactccaatttactctagaattaccctgagagcaagattggcagcaaggcttgttggtaatcgcttgatcacgaacatgacgctaactttcttcag| | GH002039.1 |
| 23 | *Magnaporthe grisea* | snR78 | gtgagtaatactttcttcttcacttcccgatcccttcttcctgatgacaaccgcaaaaccgcaaacaaactagagtctttgaccatacggaatgaaacttctatttgtcacgtcagtctgaaagaagcatcacgaactgtcatttgttttgacattggttgatgagtacagagcatttgctaatattcatcacag | BM863115.2 |
| 24 | *Magnaporthe grisea* | snR77 | gtacgttcctcttcactcatattcgttatccctcacctgatgatcaattttttgcttcggcaaagttggaattaccgactgagactcatgtcgctgaagaagcaaactggctgtttctgatttttacacctttcttcaaatttgtatgcagcatggatgtctattcagggtgctaacaaattgtgcag | BM863115.2 |
| 25 | *Magnaporthe grisea* | snR76 | gtatgcaccttcctctcaacaaccacccgaacccgtcgactcccttgcatgatgtaacggcactgggctccgatatcaaatgagcagacatgccacttctgcgacaggcctagaaaacctcgtccactgaacagaaaccgcacattcaacattttgcaccgtcaaacaggggatattcttttgctaactgcgcggcctctatctgcag | BM863115.2 |
| 26 | *Magnaporthe grisea* | snR75 | gtaagtcgattattatgatctcattttctctggatttcctgatgattctagatgacgagtcttattcgggtttccgatgcagacaacaaaacttactaccattcatgccttaactgaacaatcctctcttgcctcgaatgcaactttcagtcatggctaggatgcaaggctaacatgactatcag | BM863115.2 |
| 27 | *Magnaporthe grisea* | snR74 | gtacgttaattaaagatacacttctcctgcacgaggttcccttttatgatgatttgacaaaccaaattaagacaagcatatgtctgagcttttgcaaagatgagactgtcattcctgatttgagaacctcggcgcaactcttttgttttgtgctcatagcaatgtgctaacttgaatcaag | BM863115.2 |
| 28 | *Magnaporthe grisea* | snR73 | gtaggtgctcattttcacttcagcatcagatattcccgttcccctctttacatgatgatttaaattttatcacgacggtctacatagccaatggctttgatgagactgcccttgacaaggcacgaatctgatacacaaatttccacccatccattttggttatgtctcaactttggatagcacttttgctaacgacgtgggtctccag | BM863115.2 |
| 29 | *Magnaporthe grisea* | snR55 | gtaagttccagtcaactcgcgttcaagcgccgtcgacgagcactcaaagctgttgatgagaaaatttccataacttttcgcgatcacctgagcgaacaggaaaaaacatgcactaccatctgaagcacactcttttgaggcttttgttatttggaacttggacaagaccgctttgtactaatttccagacgcag | BU643353.2 |
| 30 | *Magnaporthe grisea* | snR61 | gttggtgcgccaatttcactttgttccttttcttttcagcctccagtctcatgatgacaacactttttttcagttctgctactgaaagcaattgatgacaaaccttataccaaaaacaacctctgaatgaactgtcaaacacactattttcacatcttcccagccatttctcaacgacgtactaatttgtgtcgcacatatag | BU643353.2 |
| 31 | *Magnaporthe grisea* | snR41 | gtgagcttcgacttccagtcttttccagcacttcccccaaccctcttgaggtgatgataaaccaaaacagggattcctcatgccgaagatcaaatgtggaatgttttctatagtttcagccttgtctgatcaagctcatatcttttcttttgagtaccaaacgggccgtgggcctacatcgtctactatgatactaacagtcgtccttcag | CD035643.1 |
| 32 | *Magnaporthe grisea* | snR70 | gtacatacgaccctttttttcgaagctcccagcgagttactactccccacatgacgaagtttgaacgaacgggcggacataatcgagcccctccttggcctttccatcaaaagcaggactgtacaaaccctgtgggaaggttgaggtagggtgctgttgatgagacctcgtcacctgatccggattctggattttgcttcgcgcgaagttacttggtgggaattcttgactaattagatcgccgacag | CD035643.1 |
| 33 | *Magnaporthe grisea* | snR51 | gtaaacaatttttgcagtacacatgtctttctctcgaatatgatgattcgttattcacactgtttgatcctgcctctggtatggaaatgaacatctttatacaaaatctgatttaatgaggtaagtcgacactgacacccagagatcttgatcgatggacacaatgctgacttgcttcgttagcctgacttttctctactttcacctcggatgatttgaaaatctttggagggttggaggctgaccccattgggatccag | CD035643.1 |
| 34 | *Magnaporthe grisea* | U45 | gtaatttactccatgcttgtgctcttttacaacattgcgatgacgaacatcaacgaacaatccaatacttacaagcaccatggggactctttttatctctagaattacgctgagcaaatcctgcgatgcagttttacatgaccattcgcactgatggcccttgctaatgctgggtcctgatcaag | CD035643.1 |
| 35 | *Magnaporthe grisea* | U55 | gtaggtcccaccaatctctacttcacccagcgtcctatcggatgattccctttgcatcaactgaacattatctacattatctacatcatctgttgatacaacccaattcaccttggagagctgattaggcctcaaacgcccatcattgacatcgatcacatgcgcttgcgccaggctaaccttctcacgggtacag | CD035643.1 |
| 36 | *Gibberella zeae* | snR41 | gttcgtacactttttcttcttttctttttctttttttcttcatctacccccctccgtatgatgatcacaacgacaccagggattcctcattcttaatatataatgtcgatactgttgtatttagtttcagccttgtctgactggagatcacaacgcacctttgcttgcttctatctatcatgaccgcattgctaactcgcgtttgtcaatag | CD458577.1 |
| 37 | *Gibberella zeae* | snR70 | gtatatttctttgccctactctttccgcgctcctcgcagccactgtatgatgaatatttgaacgaacgggcggacataaacgagtactgctgcctcccaccctagaaaagcgaggacgtacaaatccttgtggtgggtgtgtagtgggacctcttgttgacatttcgtcacctgatcgtggctcttggaaatggattcttgtcgatatctggtttaggaaccagtggctgactgttgaggatctag | CD458577.1  CN813242 |
| 38 | *Gibberella zeae* | snR51 | gtacctagacaaagaattcttgctcttgcacatcatctatccatctctttcgtgatgagttatttatcaaattgtattcacactgtttgattcggggtcttcggatcctgggacatgaacacaacgtcaacttctgactgatcggtacgctactcaccactggacttgttcaggatctcatcactaactggcctagactttatttcatgctttttttgacttcaaaaatgatgcggccatgctgactccgccgcag | CD458577.1  CN813242 |
| 39 | *Gibberella zeae* | U45 | gtaagaatcttcccctctcctgcttttcttgtttgctttttacgatgatcaacaatacatgaacaatccaaatcagacctaccgatggtgatatttctactatagaattacactgagaaagctctggacctcagaatttgcaggacaattttgtctagaacctcttactgactggtcactag | CN813242 |
| 40 | *Gibberella zeae* | U55 | gtaagacaaatatttttttacacactttcccaaccatatgatgactatgctattattagctctatccgagttcatcatgacgaacaacctattcaccttggagaactgatctggttccatttatgccttttatttaaaagatgctttagagaacctcatggctaaccagctttcgtag | CN813242 |
| 41 | *Gibberella moniliformis* | snR77 | gtacgttcatatacagcgtctcctgttgcatcctaccccgtctctcgtgatgatcatttcttttacaatatacgctggaaattaccggctgagttcttctttgacgatataaaatggctatttctgattgagactcccctcttcacacttgttgccaggcatgtagtagcttgcgctgacctaaaaaag | DR617370.1 |
| 42 | *Gibberella moniliformis* | snR76 | gtatgcgatatctcatctcgacccgcacccgacctttcatgatgaagcggcactgggctccgaatcataccacatgagcaagaaaagccactttgcggcaggcctagaaaacctcgcccactgactcgtcaccatctactttcacttgttggatgttttcgggccattgaggctaacatgtctcaag | DR617370.1 |
| 43 | *Gibberella moniliformis* | snR75 | gtaagtaaaattttacatatttcagcagcatcctcgcttcctgatgaaccctttagatgacgagtcttatacgagttttcgatgcagacacaatcctactttaccattcatgcatttcctgacacgcgattcttttacaaatttgctcgcaaacactaggcgacttgagctaaccaattttcag | DR617370.1 |
| 44 | *Gibberella moniliformis* | snR73 | gttagttttcccttgtcttctgtctcctcaatccctcttaaatgatgttacaatatcacgacggtcaacgtagccatattgctttgacgataacgcctgtgacaaggcaccttaccctgaaattcctattgcattcttctttcgatgtttatcatgcatttatgacaagtcgctaacatgactttgaag | DR617370.1 |
| 45 | *Gibberella moniliformis* | snR41 | gttggtaaataatttcctttgttttttccaaaccctctccggagtgatgatcaaaacgacaccttagggattcctcattcttaaaatactatgtggatactgttgttataagtttcagccttgtctgactggagatcacaacgcctcttttgcttgcaccgttttctactgatgcaattgctaactcgcgtttgtcaatag | DR662794  DR648230 |
| 46 | *Gibberella moniliformis* | snR70 | gtaaatctcatcttccccctcaagcgctcccagccactttatgatgaagatttgaacgaacgggcggacataaatgagtactgctgcctcccaccctagaaaagcaaggacgtacaaatccttgtggtgggtgtgtagtgggacctcttgttgatacttcgtcacctgatcgtggctcctgtggaaatggattcttgtcggtatctggtagaagaactggtggctgactgttgaggatctag | DR662794  DR648230 |
| 47 | *Gibberella moniliformis* | snR51 | gtacttgaacaaagaattcttgctcttgcatacacatccctttcgtgatgagttgtttttttagtattcacactgtttgactcttggtcttcggaccttgggatatgaacacaacatcaacatctgactgattggtatgcttctgatgctctaaccatcgcgattccatggctaacctgtctagatctgatcttgcgcttttgttgccaaactcaatgatgacggatgctaattccgtcatag | DR662794  DR648230 |
| 48 | *Gibberella moniliformis* | U45 | gtaagaatttcccccactccctgctttattttctacttcacatgatgaatatcatacacgaacaatccaaatcagaccagcacaatggtgatatagcaatctctagaattacactgagaagctttggacctcggaaccggcaggatgtttttgtcacaaacctcgtactgacccactcactag | DR662794  DR648230 |
| 49 | *Gibberella moniliformis* | U55 | gtaagacggcattttccttgcgcacatcttctgcacacccctgccatatgatgactctgctattattagctctatccgagttcatcgtgacgaacaacctattcaccttggagaactgatatggtttcctacatatgttttttttccacattgcggaagatatcatagctaaccatgtttattag | DR662794  DR648230 |
| 50 | *Aspergillus flavus* | snR78 | gtaagttcactcttgtctgacttttgtctattcttattttcttccacatcttccatcatgatgtaacctacaaccatttacaaactagagtctttgacctatttggcatgaaaactcggttttgcacgtcagtctgatctgcttgcaaagaaagctttctgctcgcttgcatttcaatacgaaagcacattgctgacttgatgtgccgcag | CO144194.1 |
| 51 | *Aspergillus flavus* | snR77 | gtacgtaccgactcctttttgtttcaccccgtctagcatgatgcttcacaacaacatatacgctggaaattaccgactgagaccttcgggttgttggcgataacacttatggcacatttctgatctgctacctcatctatctgtttgatctcttattcgcacatgtgtgattgtatgctgacggtcaaaag | CO144194.1 |
| 52 | *Aspergillus flavus* | snR76 | gtaagactcgaccatcctatttccctacatcattttcgaagatgaaatggcactgggctccgacggcgtccgacagacgaccggtcttgatggatatgccttttttgcggcaggcctagaaaacctcgcccactgagatcatacaactacacaaatatattgatcctacaagacttgggcctggggctaatggtcatggatag | CO144194.1 |
| 53 | *Aspergillus flavus* | snR75 | gtaaatatcaacttgtctcatagctctcaagcctcacatgatgatttcctagatgacgagtcttattcgagttatcgatgctgacaccatcttttttttaccattcatgcatttctgactcccacctttggaataagtttcaacggagtttctgtgaacgttgctaactctgttgccatcacag | CO144194.1 |
| 54 | *Aspergillus flavus* | snR74 | gtatgtaatcacctcgccccgaatccagttctcttcgacatcatgatgaatcgacattaaaacaaatgacaagcatatgtccaagtcagcattgctggcgaggacgacaactgtcaggactgatcagtcaacaacttgtcttcgtctgtcacagccttgaagcaatcgaagctgattgtatgtgcag | CO144194.1 |
| 55 | *Aspergillus flavus* | snR73 | gtaagttgaatctgcttttcttcgagttcacgctgcattgatacctgtacaatgatgattttcttttattttaatcacgacggtcaacataatcaaatacattgatgaccaccgcctgtgacagggcatttaatctgaaataccctccatcagttcgatttgtcagttttcaggaattgattgttccagtgctgactctggctttag | CO144194.1 |
| 56 | *Aspergillus flavus* | snR41 | gtatgcgacagacttttctctcgtttatttcttttcttcccctcgaatgatgacgcttatgacacgctatggcattcctcaaacttaatcaccatgtggaattcgtgttttaactcaagtttcagccttgtctgacatgctttacatcttttgctgctatcaccaagtttaacttgcatgatcattgactaacagacttgtcaatag | CO147811.1 CO135189.1  CO143673.1 |
| 57 | *Aspergillus flavus* | snR70 | gtacataaatttcttccattttcttgttcgattccaatgaagaaaccatgaacgaacgggcggacataacgtatacatctacctgcatccatcaaaagcagagcgtacaaactttgcgggtgtaggacggtgtactacttgttgagaactcgtcacctgagctttcggcttatccttctttggttaccatccgtgggacttgcaatttactaacaagtcttcag | CO147811.1 CO135189.1  CO143673.1 |
| 58 | *Aspergillus flavus* | snR51 | gtattgtcacatttttctgttgtttctgttgtgcacacccaaatcagtcaactttatatgatgaatctctttattcacactgtgtgatccatgctctgcatggctatgaactttatacaaaaattctgatttaatgaggtgagtttgatatgctcttgtgatgcaagtaccattatgctaacatttattagcctgactattgttgacacatcacacttctgaatcacacataatactttctcgatatgtgctgactcgtttcgtgatgtag | CO147811.1 CO135189.1  CO143673.1 |
| 59 | *Coccidioides immitis* | snR78 | gtgagtgatgcccctttatcatttttcctagcaatatcttctatccttaactttgtgatgatttgtaaaaccgcttaaactagagtctttgaccttgtggtatgaaaattgcatttgcacacgtcagtctgatctcgaagaagaccctggaggaattgatttctttcctttgaattcctttctccatgaaacatagctaacctactcgaag | GH354057.1 |
| 60 | *Coccidioides immitis* | snR77 | gtatgtcgtcaacatattaaacggggccaagctccgacccataacatgatgattctttctttgtaactatacgctggaaattaccgaccgagaccttcgggtctctgacgatacaaaatggctgatatctgatttcacaccttcaccgtttttcctgctcagtatcgcatgttttgaactgttgactgacaggtatatacacag | GH354057.1 |
| 61 | *Coccidioides immitis* | snR75 | gtaggtgctccttatttattattacatctccgctcctcatgatgatctcttagatgacgagtcctattcgagtactcgatgcggacaacctttctttttaccattcatgcatttctgaaccccctatgttgattttccattttgataactttcggcctcggggttactgacgtgaagacag | GH354057.1 |
| 62 | *Coccidioides immitis* | snR74 | gtacgtgcctctattgctgttgacaactcgacactctttcatgatgagccaagactataaaataagacaagcatatgtccaagctggcaattgtcggcaacgacgacagcgtcattccctgatactgttggaccatttgacatggtgttccatcctgtcgcatacaatgtttagctgattttgcggtag | GH354057.1 |
| 63 | *Coccidioides immitis* | snR73 | gtaagatattcttggccttgatattttgcgttcttctcctatgatgattttatttttttttatcacgacggtcaacatagcttttgcattgatgaccacagcctgtgacagggcatctatctgataatgtccacaattcgttcattgttccgggttacacttgtcatacaaattactgacttgggctcggcgcag | GH354057.1 |
| 64 | *Coccidioides immitis* | snR41 | gtatgaacttttcccatacacgcctgttcccattccccttgattatgatgactattacgacatgcaaaggcattcctcaaacttaatcaccatgtggaatctgtcatgttatcaagtttcagccttgtctgatcaaaaaatacatttgtgctacttttatgtttcctctcttgaggcaactcttatacttgatcggctatagatgggtttgctgacgtggccttccattgtag | GH362268.1 |
| 65 | *Coccidioides immitis* | U55 | gtatgtagtctggaaaactttcctttttgcctggattccttccccaattcgtatcactcgcccctgcctgcatgatgaactcttaatttcgctgtgttcttaccgaggcacactttgaagaaacaaaatatcaccttggagaactgacacctactttctatcgaagtttatcggaaattatgtcactgccttgatgtttcttatgctaatggccatttctag | GH362268.1 |
| 66 | *Sclerotinia sclerotiorum* | snR78 | gtaagcgcatctatttcttctccccctttcattcattatgtgatgacaaaacaacaaccgcaaactagagtctttgatcctacgggacatgaaacttttgtaacacgtcagtctgatattggatcacaattttgctcattttagcaacttgataactttgatcattgctaacaattgagacag | CD646280.1 |
| 67 | *Sclerotinia sclerotiorum* | snR77 | gtaagctccccctatttccattcttttctctttttcccacatacaatttgtgatgatgatcagtttgtgctttgcacgctggaaattaccgactgaagtcctcgggcttttgacgacaaaaaaggctgttactgagcaaattcttccaactcaattttgattaatattttttcctcttaagttgatgctgactatgttataag | CD646280.1 |
| 68 | *Sclerotinia sclerotiorum* | snR76 | gtacgactcaattctcctcacttttcaacaccacgattgcacaaaatgatgaatggcactgggctccgacagttccaaaatggttctggtcttgatggatatgccattttgcggcaggcctagaaaacctcgcccactgatttcagtcatcttatcatcatctgttgtacacatgtttgaaatacggtttctgattttggaagcatag | CD646280.1 |
| 69 | *Sclerotinia sclerotiorum* | snR75 | gtaaataaacattttctcttgccctccatactttttatgatgagcatttttagatgacgagtctgattggagttttccatgttgacaacatcaaaattaaaccattcatgccttctgaatttttttagataggaatccttttttaatgtgtatcgtgtcaaagcatgtgctaatatcaacctttttag | CD646280.1 |
| 70 | *Sclerotinia sclerotiorum* | snR74 | gtaagaaaacctcagtacatttctccggcagccctcgagcattctgatgacatcgatcgacaaatgacaagcatatgtccaagcctcacggttgtgacgagcctatcttaactgaatatatgctcatgccattatatacttttgttatcaagtcatgaccattgctaacggttatcgttcag | CD646280.1 |
| 71 | *Sclerotinia sclerotiorum* | snR73 | gtatgttactattatctcttatctattttcatcccttcacctttttatatgatgattattttatcacgacggtcgacatatccacacgttggtaattgatgaaaatgcctatgactaggctttgatctgaaatttcatttctgtccacataatctaaataattttcttcgcccatgaaaccattgctgacgattcaaag | CD646280.1 |
| 72 | *Schizosaccharomyces pombe* | snR78 | gtatgtgtctataaccacagatggaatctcctgatctttgatgaccattttttaaaatacaaactagagtttctgattaatttatgatttcaaattcttgctgagtttagttgtttccttctgtattttggttatttcaacacttatgctaacataatttag | Our previous work |
| 73 | *Schizosaccharomyces pombe* | snR77 | gtaagtgtttgaaagaattgtttatgttgtgttggaaaagatgaaaaaaactttttttggaattaccggcctaggaatagtttactcctattccgctgaagaataaatgtgctgcaattatgctgatccattacaatatcacaactttttttatgggattttcctattattaacatcattag | Our previous work |
| 74 | *Schizosaccharomyces pombe* | snR75 | gtatgtgatatgagaaaaattgttctcaatgaggaataattagatgacgagtctgattcaatatgaagaaaagactcttaaaattccattcatgctaactgagagaacaaatttttaacctaaatctatgccaatattttactaacataattatag | Our previous work |
| 75 | *Schizosaccharomyces pombe* | snR74 | gtatccaaaacttaattgaaaactagacttcaaatatgtactttagggtattattaggatgatttattaaatttcaaagacaagcatatgtctgatgccattggttttgaagaagtgaaatgcgtctgattatagacaacctaaagttatatatttccatgtcctaagttttcgtgttaaaacaacttttctaacgtatttgcaag | Our previous work |
| 76 | *Schizosaccharomyces pombe* | snR73 | gtaagtagatcagcttgaggtcgaattttagttcacatgatgtttttactttaatcacgacggtctacgtagtttctacattgacgaagattgcctgtgacaaggcgctttttctgaatgtactttatatatcgacctctaatttttagtgtcggttcgtttattaacattttttag | Our previous work |
| 77 | *Schizosaccharomyces pombe* | snR57 | gtatgtgcaaatttttttttttttaaatatgttttgagttgatgatgatcatttctgttcctagtagggcattcgatgcggacacttttgtattttaggaattcttcgttgctgaactaactcaataattttgaaagcctaattcgattactattaattactaacttcttag | Our previous work |
| 78 | *Schizosaccharomyces pombe* | snR55 | gtatgtttattgagtctgtgttaagattgctaagatgattatactttacccctttctgttggtgataatcaatgatcctcagaaaacatgcatcaccgaatgaacatagcacgtctcagccattaatttcgtaattattaatgtttttatattaactaatttag | Our previous work |
| 79 | *Schizosaccharomyces pombe* | snR41 | gtaagcataatttaaaaacagaagttcctgtagctttactactaacaatcattgtgatgataaaacgacgaaaacagctgaattcctcatgttgaattcgttatattattttttaagtttcagccttgtctgatcatgattttttttacttcttttttctttatttttttgttattttttctgacgtttttag | Our previous work |
| 80 | *Schizosaccharomyces pombe* | snR70 | gtaagttaaaaattttaggttgcttttcaagtttaaggatgatacctaagacgcaacgggcggactaaatgggttactacaatgcttcaactgtacaatgaagttgtaactcacatgttgttatcgtgtcttactgaaatacttgattagcattaaattttgtcttttttttttttgtttgtatctaaagtattaactaag | Our previous work |
| 81 | *Coprinopsis cinerea* | snR78 | gtgagtctcttttcccaatcgtttgcagcttgcaacgttgcctagtgcgatgatcccaatcaattcaaactagagtatctgagcgtcaacgacgccatgcttttatcatgctcatttctgatcccaccttacctgctgtttttgaaaattttactgaccttatttctcag | AACS02000004.1 |
| 82 | *Coprinopsis cinerea* | snR55 | gtaagcctcgtttcgtctttatcgctcgaatttattgttcctttcgtttgctttgatgatattctcccttttctcgaccggtgacttttggtatgagccattgtcgaaacatgcatcaccaactgagccctgcaactttctgaaatcgctatcaacagttttactgacgaatatctcctttatag | AACS02000011 |
| 83 | *Ustilago maydis* | snR57 | gtaagtacgctctcctcagcttgttgctcagccctctcgcctttcacaggagttttccgagatgatacttttaactattgtgctttacacgaattgaccgagtcgcatcgggacatagtagtggtgggggaaatacaataatggaattcttcgttgctgagatccttcctgttcccaagacgacagacatgggccgaccgcaacacgtacaacagcgcatcctcattgcaagtgtgctgaccgatccgctttgcatctctttgtttcttttgcgtttctttcag | EH033051.1 |
