# Supplementary material for: Splicing-Related Features of Introns Serve to Propel Evolution
Source: PLoS One. 2013 Mar 13;8(3):e58547. doi: 10.1371/journal.pone.0058547 (PMC3596301; doi:10.1371/journal.pone.0058547)
Supplement: Table S2 — snoRNA-associated introns and adjacent exons. Intronic sequences are set in lowercase letters and flanking exon sequences in capital letters. snoRNA sequences are in gray and short direct repeats sequences are in red. (DOC) [file pone.0058547.s016.doc]

| **No.** | **species** | **snoRNA** | **Intron and adjacent sequences** |
| --- | --- | --- | --- |
| 1 | *Schizosaccharomyces pombe* | snR72 | ACTTGTGAATTTAGTATATGTTATACTTTTAAGgtaagttttctactgttagaattgacgtttggaaaagtaaatggagaaaggatttctttgatgattccaaaaaagcatagttcaactgagcatcgtatgctgaaactttttccatctgatttgcacgtcagtctgagatttctcgttcccttttgtttctttctttttcaacgtctgtttgtctgacttttgtgttggctgttttactgacccatactttaagGTGTTCTATATTTTTTCAAGGATATCATTA |
| 2 | *Schizosaccharomyces octosporus* | snR72 | GGATTTATTTAGATCCAAATACTTTATGAACAGgtatgtcgttcagtaagtatagcaaagagaagttgttttatttgtttttctttatgcagaaaattctttgatgatctgaatatgcatagttcacgctgagcttactatgctgatacataattcactgatttgcacgtcagtctgagaatttagatcagcttcttttgctttccttcattctctttctcatatgctaacaaatatttttta-gAAGGATTAGATTCGTTTAGAGTTTGTTAATATTCATTTATGTAC |
| 3 | *Schizosaccharomyces cryophilus* | snR72 | AGTTCTACTAGCTCTAAGAATTTTTGGAACAGgtaagttgtttaaaaaattgcagatagaagttggattttttttattttacataaaaagaaagaaaaaaaaactaaattctttgatgatttgaaaatgcatagttcacgctgagcttattatgctgatccttattcattgatttgcacgtcagtctgagagtttggatcaacttcttttgcttttctttttccctttttttcaaaatgctaacactatttagAAGTAAATAGATTGGTTAATATTT |
| 4 | *Schizosaccharomyces japonicus* | snR72 | CTCCATTCCACCATCAACCTTCAAGCTTGgtaagttcaatacagcttctccattggaattgcaatgatgataccataaaagcatagttcagctgagtgacaagcatgctgatactccaatccatcgatttgcacgtcagtctgagtcaattccgatgagaagcactgtttttctcttcgaatcaatttactaacatctgcaggtttataagTTAGACGGCCAATAAATATTCTGACATGATAGAGTGTCAATTTAAGTATGCATTG |
| 5 | *Candida parapsilosis* | snR72 | CATTTCCAGTTAATCTTCAAAATTGCTTTAATGCAATCAAGTCGTGACTAAATGgtatgttgat-agcatccgagcagttattgctctttcaaacattctcctccaagtttcgtctacaaaaatatgatgtcttttaattcttacatatcatgagggggtactgcctccgatgatatacaaaaactaaaaacgatttgcatgtcagtctgatttttatagcacaatttgcggagaatattgagtattgtttggagtattatttgaaaagtttactaacagttttgattagATTTGGAGAGTATTGAGGTAACGTAGTTCTGGAGTTAAGTTGGTTTC |
| 6 | *Laccaria bicolor* | snR75 | GCGGAAAGCCTTTTCTTGGTTTCTTCTAGCGAAGTTTTGGACAGgtcagttccgcatcttatcgatttcttttatgatgagatatttagatgacgagtctgaaccatgataggcagagtttctctgtctgtctgaacatgacgactcaactaaaattataccattcatgcttttctgatttttttcttgcttttttgacacccatctgttacccgagagcgcccaagctgatttttgccaacagGTAACATCATACACCATGCCTATACAAGAAGACGATTGGTCCGACTCTGAC |
| 7 | *Coprinopsis cinerea* | snR55 | TTGTCGTCGTTGAGAGAACGATCCCAGCACTCAAGgtaagcctcgtttcgtctttatcgctcgaatttattgttcctttcgtttgctttgatgatattctcccttttctcgaccggtgacttttggtatgagccattgtcgaaacatgcatcaccaactgagccctgcaactttctgaaatcgctatcaacagttttactgacgaatatctcctttatagGTAGAACCGATGCATAGGAGCATGGTGTGAGTTGAA |
| 8 | *Aspergillus nidulans* | U45 | GCTTCTTGCCTCTCTGATTCTGTTTC--TAAgtgagtatactctcgtgcccctgcatctgcttgtcttcagggatgaattcctttcaggatatccacggccgaaaaaacatgaaggacctcttcaaagactctagaattactctgactcatgaagacaactgcaattgataaagtcggcgtctgctgtatccgttgctaatatctcgcttgtccagGTTTCCCTAAACTTGTACGACGACTAATACTTGATCATCA |
| 9 | *Gaeumannomyces graminis* | U45 | TCGTGTTTGCCAGGCGCCCCGAAAAGgtaattgccttctccttctttctttcgagcttcgcccctttgcgatgatgaccaaacaatcacgaacaatccaaatcttaccagcattatggggactacaattactctagaattaccctgagcaaagcacacacaaccttgtcttcttctgtagcccgttgggctagggcgagcttcttactgacatgccgagcctctagGTAATCCGCTAAATGTCCAT |
| 10 | *Gibberella moniliformis* | U45 | TTGCCCAGCTTTTGCGCGACCATGACCTTGAATTTCTCAC-AGgtaagaatttcccccactccctgctttattttctacttcacatgatgaatatcatacacgaacaatccaaatcagaccagcacaatggtgatatagcaatctctagaattacactgagaagctttggacctcggaaccggcaggatgtttttgtcacaaacctcgtactgacccactcactagTTTGTTTGCTCACCTGTTAGCCGTTGA |
| 11 | *Alternaria brassicicola* | U55 | CGAGTCTACCAGCCTGCACCTTTCGgtaagttcagccacatcccacattacccgccacctcttgtatgatgaacaacatgatttcgctgtgttctttcttaattacattgaagatacaatcaccttggagaactgacccctacaccttattgtcatcacttgctcgcacacctgggttggactggtttgctaaccacgtacagTGATTT-GGTCCGGTTGCGTGCCTTGGA |
| 12 | *Aspergillus fumigatus* | U55 | GATACCTGGATTGTAGTGACTGCTAACAACATTTACAGTgtgagtgctacccatactcttttgccatggatgttgtatgatgatctcttgattcgctgtgttcttactgaatacatcttgaagaaaaacatcatttcaccttggagaactgatacctcgtcttctctgatcgtcaatcttggaaccctgaaagtagatgtggctaacctgtcatttgcagACGATTACTCAGTTACATATCCTAGGGAAATGCA |
| 13 | *Neosartorya fischeri* | U55 | CGTAGTGATTGCTAACGACATTTGCAGTgtgagtgctacccctactcttttgccatagatgttgtatgatgatctcttgattcgctgtgttcttactgaatacatcttgaagaaaaacatcatttcaccttggagaactgatacctcgtcttctctgatcatcaatcttggaaccctgaaagttgctgtggctaacttgtcacttgcagACGATTGCTTCGTTATAG |
| 14 | *Aspergillus nidulans* | U55 | AAACAGCGTAGCATTAATCATTTACTGAATAGAgtgagtattccttctgcctgctttcccttatgatgatgatttctttaattgctgttacttctgaatgcattttgaagaaacttctttttcaccttggagaactgatgtcttgccactcctgaacttctcaccctgttttttgtttttttcattgctgaccaattcaaatagTTATGA |
| 15 | *Botryotinia fuckeliana* | U55 | CATGACCACGTCAACAGGTTTTTCACAAAATgtaagccacttctccactttctatcctcacacattttgctctgatgaacccaaatattcgctgtgttctttccgagtcaacctttgacgaaacacatatttcaccttggagaactgagatagtactcatgttactcatttccaatcgtatatcaacgaaggttgggacttgaagctaacctgcatagACATAATGGTAATTTAAAGCTGGAGCTCAATTTGGAA |
| 16 | *Epichloe festucae* | U55 | CCCGCCTCCTCATACCCTATCAATGgtaagcactttgtcgtctttttttttttataatttcaccccgccattaggatgacttctttattagctctagctgagttcatcgtgatgaacaaccaacctactgtttcaccttggagaactgattaggctctattttgctctatcgtgattgctgcaatgttgtccccaagactctggctaatacagtcgcctgaatagATTCACACAATGGTCGACCTGGAAGCAGAGACGAAAA |
| 17 | *Grosmannia clavigera* | U55 | GTAACCCGTGTTGTTTTGACAGCCGCTTCGACGATATTACgtaagcatatatttctaccctctcgctgccactaagatgatacacctactgcatttttgtctgattcatttgatgagacctttttacctattttcaccttggagaactgatgtggcctctaacccctgctacttattgattgtgcccctgtagcacagatgctgactgcgtccagGATC-TACGACTCGTTTGCATAGCATAGTTTGCCGCAAT |
| 18 | *Magnaporthe grisea* | U55 | TCGCTGTGATCATGGCGCCCATGTGAATCATTGgtaggtcccaccaatctctacttcacccagcgtcctatcggatgattccctttgcatcaactgaacattatctacattatctacatcatctgttgatacaacccaattcaccttggagagctgattaggcctcaaacgcccatcattgacatcgatcacatgcgcttgcgccaggctaaccttctcacgggtacagAT-ATTGCTTGTTGATCGACCATGC |
| 19 | *Sclerotinia sclerotiorum* | U55 | GAAGCTATTGGTAAAGTTGTGTAAAACGAAATgtaagctacttcttttcattcacctccacactctttgctctgatgaacccaaatattcgctgtgttctttccgagtcaacctttgacgaaacacatatttcaccttggagaactgagatagtacccatgttattcatattcaaccttctgtcaacgatagttagaacatgaaactaacttgcacagACATTTTGAAATTCGGAATCGAATTTAATTTAAGTTGAAGCAATAGAAGTTGTAGAT |
