# Supplementary material for: Splicing-Related Features of Introns Serve to Propel Evolution
Source: PLoS One. 2013 Mar 13;8(3):e58547. doi: 10.1371/journal.pone.0058547 (PMC3596301; doi:10.1371/journal.pone.0058547)
Supplement: Table S3 — Exon loss in fungi. Intronic sequences are set in lowercase letters and flanking exon sequences in capital letters. Conserved 5’splice canonical sequences are in blue, branch-point sequences and the 3’splice canonical sequences are in red. (DOC) [file pone.0058547.s017.doc]

| **No.** | **Species** | **Paratactic introns** |
| --- | --- | --- |
| 1 | *Candida albicans* | AATGgtatgt..snR41..tgctaacatatttgtccaggtatgt..snR70..ttctaacattaacttttttttggcagATT |
| 2 | *Candida dubliniensis* | ATAGgtatgt..snR41..tgctaacatatttgttaaggtatgt..snR70..ttctaacattattttttggagTAG |
| 3 | *Clavispora lusitaniae* | AATTgtatgt..snR77..tactaacttttaggtatgt..snR76..ttctaacagtttagCAT |
| 4 | *Clavispora lusitaniae* | AAAGgtatgt..snR73..tactaacttttaggtatgt..snR72..tactaacaatcacgtagAAT |
| 5 | *Clavispora lusitaniae* | AACGgtatgt..snR57.. aactaatacggttcaggtatgt..snR55..aactaacaccttagCTG |
| 6 | *Clavispora lusitaniae* | AATGgtatgt..snR41.. cactaacattataaggtatgt..snR70..aactaacaatagTAG |
| 7 | *Candida tropicalis* | AAAGgtatgt..snR41..tactaacttatttcaggtatgt..snR70..aactaacaatttataacaaatagTTG |
| 8 | *Debaryomyces hansenii* | ATAAgtatgt..snR77..tactaacaataatcttaggtatgt..snR76..tactaacaaatttcaatagACA |
| 9 | *Debaryomyces hansenii* | AAAGgtatgt..snR41..tgctaacattaaatttaggtatgt..snR70..cactaacgcccaatagTCT |
| 10 | *Pichia guilliermondii* | ATAGgtatgt..snR77..aactaacaataggtaagt..snR76..aactaaccacaagTCA |
| 11 | *Schizosaccharomyces cryophilus* | AAAGgtatgt..snR77.. tgctaacggcttttaggtatgg..snR76.. ttctaacctcaagTTA |
| 12 | *Schizosaccharomyces japonicus* | AAAGgtaatt..snR77.. tactaactcgtctaggtatgc..snR76.. ttctaacatctacagTCG |
| 13 | *Schizosaccharomyces octosporus* | AAAGgtaagt..snR77.. ttctaacattctattaggtatgg..snR76.. ttctaactattgctagTGT |
| 14 | *Schizosaccharomyces pombe* | AATGgtatgt..snR57..tactaacttcttaggtatgt..snR55..tattaactaatttagATT |
| 15 | *Yarrowia lipolytica* | AACGgtaagt..snR77..tactaactccaggtatgt..snR76..tactaacctcagTTA |
| 16 | *Yarrowia lipolytica* | AATGgtgagt..snR57..ttctaacatcaggtgggt..snR55..tgctaacattagTTA |
| 17 | *Yarrowia lipolytica* | AAAGgtatgt..snR41..tgctaacctcaggtgaga..snR70..cgctaacaacagATT |
| 18 | *Ajellomyces capsulatus* | AAGgtatat..snR41..tgctaac..taggtatac..snR70..cactaac..taggttaag..snR51..cgctaat..tagGGA |
| 19 | *Ajellomyces dermatitidis* | AAAGgtacgt..snR41..tgctaac..taggtatgc..snR70..aactaac..taggtaaat..snR51..cgctaat..tagGGA |
| 20 | *Alternaria brassicicola* | AAAGgtaaga..snR41..tgctaac..caggtacat..snR70..agctaac..taggtatga..snR51..agctgac..cagTTG |
| 21 | *Arthroderma benhamiae* | AAAGgtgtgt..snR41..tgctaac..taggtacgc..snR70..tgctaac..caggtatat..snR51..tactaat..tagGTT |
| 22 | *Arthroderma gypseum* | AAAGgtgtgt..snR41..tgctaat..taggtatgc..snR70..cactaac..caggtatat..snR51..tactaat..tagGTT |
| 23 | *Arthroderma otae* | AAAGgtgcgt..snR41..tgctgat..taggtacgg..snR70..tactaac..caggtatat..snR51..tactgac..tagGTT |
| 24 | *Ascosphaera apis* | AAAGgtaaga..snR41..gactaac..taggtacat..snR70..gactaac..taggtattg..snR41..tgctaac..cagCCT |
| 25 | *Aspergillus clavatus* | AAAGgtatgc..snR41..gactaac..taggtacat..snR70..gactaac..taggtattg..snR41..tgctaac..cagCCT |
| 26 | *Aspergillus flavus* | AAAGgtatgc..snR41..gactaac..taggtacat..snR70..tactaac..caggtattg..snR51..tgctaac..cagCCT |
| 27 | *Aspergillus fumigatus* | AAAGgtatgc..snR41..gactaac..taggtaaat..snR70..gactaac..taggtactt..snR51..tgctaac..cagTGT |
| 28 | *Aspergillus nidulans* | AAAGgtaagc..snR41..ctttgac..taggtaaac..snR70..tgctgac..taggtattg..snR51..tgctgac..cagTAG |
| 29 | *Aspergillus niger* | AAAGgtatgc..snR41..tgctaac..taggtaaat..snR70..tactaac..caggtactt..snR51..tgctaac..tagGGA |
| 30 | *Botryotinia fuckeliana* | AAAGgttcgt..snR41..agctaac..taggtacat..snR70..tgctaac..taggtaaat..snR51..tgctaac..tagCCT |
| 31 | *Chaetomium globosum* | AAAGgttagt..snR41..cgctaac..caggtatgc..snR70..aactgac..caggtaata..snR51..tgctgac..cagGTA |
| 32 | *Coccidioides immitis* | AAAGgtatga..snR41..tgctgac..taggtatgc..snR70..tgctgac..taggtacgg..snR51..tgctaac..aagCCT |
| 33 | *Coccidioides posadasii* | AAAGgtcagt..snR41..tgctgac..taggtacat..snR70..gactaac..taggtaata..snR51..cactaac..cagATA |
| 34 | *Epichloe festucae* | AAAGgtcagt..snR41..tgctgac..taggtacat..snR70..gactaac..taggtaata..snR51..cactaac..cagATA |
| 35 | *Fusarium oxysporum* | AAAGgttggt..snR41..tgctcac..taggtaaat..snR70..ggctgac..taggtacct..snR51..cgctaac..tagGTA |
| 36 | *Gaeumannomyces graminis* | AAAGgtaagc..snR41..tgctaac..caggtacat..snR70..tgctgac..caggtaata..snR51..tgctaac..cagGTA |
| 37 | *Grosmannia clavigera* | AAAGgtaaga..snR41..tgctaac..caggtactt..snR70..tgctaac..caggtacta..snR51..tgctaat..cagGTA |
| 38 | *Gibberella moniliformis* | AAAGgttggt..snR41..tgctaac..taggtaaat..snR70..ggctgac..taggtactt..snR51..ggctaac..tagATC |
| 39 | *Gibberella zeae* | AAAGgttcgt..snR41..tgctaac..taggtatat..snR70..ggctgac..taggtactt..snR51..tgctgac..cagATG |
| 40 | *Magnaporthe grisea* | AAAGgtgagc..snR41..tactaac..caggtacat..snR70..gactaat..caggtaaac..snR51..ggctgac..cagGTA |
| 41 | *Magnaporthe oryzae* | AAAGgtgagc..snR41..tactaac..caggtacat..snR70..gactaat..caggtaaac..snR51..ggctgac..cagGTA |
| 42 | *Nectria haematococca* | AAAGgttagt..snR41..tgctaac..taggtaatg..snR70..ggctgac..caggtacct..snR51..tactaac..tagGTA |
| 43 | *Neurospora crassa* | AAAGgtttgt..snR41..tgctaac..caggtatgc..snR70..gactaac..caggtaata..snR51..cgctaac..cagACT |
| 44 | *Neosartorya fischeri* | AAAGgtatgc..snR41..gactaac..taggtacat..snR70..gactaac..taggtactt..snR51..tgctaac..cagCCT |
| 45 | *Paracoccidioides brasiliensis* | AAAGgtacgt..snR41..tgctaac..taggtatac..snR70..aactaac..taggtataa..snR51..tactaat..tagTGA |
| 46 | *Penicillium chrysogenum* | AAAGgtaagg..snR41..agctaac..caggtaaat..snR70..ttctaac..taggtaact..snR51..cgctgac..aagTGA |
| 47 | *Penicillium marneffei* | AAAGgttcgt..snR41..agctaac..taggtaaac..snR70..aactaac..caggtacat..snR51..tactaat..tagATA |
| 48 | *Phaeosphaeria nodorum* | AAAGgtaagc..snR41..cactaac..caggtacat..snR70..aactaac..aaggtacgg..snR51..tgctaac..cagGCT |
| 49 | *Podospora anserina* | AAAGgttcgt..snR41..tgctaac..caggtatgc..snR70..gactaac..aaggtaatt..snR51..cgctaac..cagCCT |
| 50 | *Pyrenophora teres* | AAAGgtatgg..snR41..tgctaat..caggtaaat..snR70..atctgac..taggtatga..snR51..cgctgac..cagGC |
| 51 | *Pyrenophora tritici-repentis* | CAAGgtacgg..snR41..tgctaat..caggtaaat..snR70..atctgac..taggtacga..snR51..cgctgac..cagGCA |
| 52 | *Sclerotinia sclerotiorum* | AAAGgttcgt..snR41..agctaac..taggtacat..snR70..tgctaac..taggtaaat..snR51..cactaac..tagCCT |
| 53 | *Sordaria macrospora* | AAAGgtttgt..snR41..tgctaac..caggtatgc..snR70..gactaac..caggtaaca..snR51..ggctaac..cagTTC |
| 54 | *Talaromyces stipitatus* | AAAGgttcgt..snR41..agctaac..taggtaaat..snR70..aactaac..caggtacat..snR51..tactgat..tagGTC |
| 55 | *Trichoderma atroviride* | AAAGgttagt..snR41..tactaac..taggtactt..snR70..agctaac..taggtacat..snR51..gtctgac..aagTGT |
| 56 | *Trichoderma equinum* | AAAGgtgtgt..snR41..tgctaac..taggtacgc..snR70..tgctaac..caggtatat..snR51..tactaat..tagGTT |
| 57 | *Trichophyton reesei* | AAAGgttggt..snR41..cgctaac..caggtaatt..snR70..ggctaac..taggtactt..snR51..agctgac..aagCAG |
| 58 | *Trichophyton rubrum* | AAAGgtgtgt..snR41..ttctaac..caggtacgc..snR70..tgctaac..taggtatat..snR51..tactaat..tagGTT |
| 59 | *Trichophyton verrucosum* | AAAGgtgtgt..snR41..ttctaac..caggtacgc..snR70..tgctaac..taggtatat..snR51..tactaat..tagGTT |
| 60 | *Trichoderma virens* | AAAGgttggt..snR41..tgctaac..caggtaatt..snR70..agctaac..taggtactt..snR51..agctaac..tagATG |
| 61 | *Uncinocarpus reesii* | AAAGgtatga..snR41..tactgac..aaggtatga..snR70..ggctgac..caggtatga..snR51..tgctaac..tagGCT |
